# Built‐in RNA‐mediated chaperone (chaperna) for antigen folding tailored to immunized hosts

**DOI:** 10.1002/bit.27355

**Published:** 2020-05-02

**Authors:** Young‐Seok Kim, Jongkwan Lim, Jemin Sung, Yucheol Cheong, Eun‐Young Lee, Jihoon Kim, Hana Oh, Yeon‐Sook Kim, Nam‐Hyuk Cho, Seongil Choi, Sang‐Moo Kang, Jae‐Hwan Nam, Wonil Chae, Baik L. Seong

**Affiliations:** ^1^ Department of Biotechnology College of Life Science and Biotechnology, Yonsei University Seoul Republic of Korea; ^2^ Department of Biotechnology The Catholic University of Korea Bucheon Republic of Korea; ^3^ Division of Infectious Diseases, Department of Internal Medicine Chungnam National University School of Medicine Daejeon Republic of Korea; ^4^ Department of Microbiology and Immunology, Department of Biomedical Sciences Seoul National University College of Medicine Seoul Republic of Korea; ^5^ Department of Biochemistry and Biophysics Stockholm University Stockholm Sweden; ^6^ Center for Inflammation, Immunity & Infection, Institute for Biomedical Sciences Georgia State University Atlanta Georgia

**Keywords:** chaperna, chaperone, influenza virus, MERS‐CoV, monoclonal antibody

## Abstract

High‐quality antibody (Ab) production depends on the availability of immunologically relevant antigens. We present a potentially universal platform for generating soluble antigens from bacterial hosts, tailored to immunized animals for Ab production. A novel RNA‐dependent chaperone, in which the target antigen is genetically fused with an RNA‐interacting domain (RID) docking tag derived from the immunized host, promotes the solubility and robust folding of the target antigen. We selected the N‐terminal tRNA‐binding domain of lysyl‐tRNA synthetase (LysRS) as the RID for fusion with viral proteins and demonstrated the expression of the RID fusion proteins in their soluble and native conformations; immunization predominantly elicited Ab responses to the target antigen, whereas the “self” RID tag remained nonimmunogenic. Differential immunogenicity of the fusion proteins greatly enriched and simplified the screening of hybridoma clones of monoclonal antibodies (mAbs), enabling specific and sensitive serodiagnosis of MERS‐CoV infection. Moreover, mAbs against the consensus influenza hemagglutinin stalk domain enabled a novel assay for trivalent seasonal influenza vaccines. The Fc‐mediated effector function was demonstrated, which could be harnessed for the design of next‐generation “universal” influenza vaccines. The nonimmunogenic built‐in antigen folding module tailored to a repertoire of immunized animal hosts will drive immunochemical diagnostics, therapeutics, and designer vaccines.

## Graphical abstract

We present a potentially universal platform for generating soluble antigens from bacterial hosts, tailored to immunized animals for Ab production. A novel RID docking tag derived from the immunized host, enhance the solubility and robust folding of the target antigen. Built‐in RNA‐mediated chaperone platform derived from the immunized host only elicited antibody responses to the target antigen and greatly enriched and simplified the screening of hybridoma clones of monoclonal antibodies (mAbs).

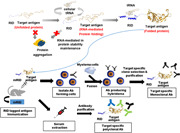

## 1 INTRODUCTION

Since the seminal work on hybridoma technology by Kohler and Milstein in 1975, mAbs have been used in a variety of applications, including enzyme‐linked immunosorbent assays (ELISAs), flow cytometry, immunoassays, and therapeutics (Givan, 2001; Reichert, 2008). Despite the central importance of immunochemicals in the biotechnology, the persistent quality issues that plague research antibodies, including insufficient specificity and sensitivity, are alarming and often result in false findings and wasted research expenses and efforts (Goodman, 2018; Weller, 2016). For instance, fewer than half of routinely used antibodies recognize their specific targets calling for further standardization and improvements in antibodies for immunochemical analyses (Baker, 2015). The quality of antibodies—specificity, affinity, and so forth—heavily relies on that of antigens including their purity, solubility, assembly status, and conformation. The quality of recombinant antigens are often compromised, rendering the screening of mAbs time‐consuming and expensive, and the “weeding out” of nonspecific hybridomas at the earlier phase of screening is important for the identification of mAbs of desired specificity.

Soluble antigens aimed to generate specific antibodies against a virus can be produced from various recombinant hosts. Eukaryotic hosts comprising mammalian or insect cells are usually preferred because of their intrinsic ability to ensure proper folding of antigens. However, the yield from eukaryotic hosts is usually low and requires expensive cell culture facilities. Bacterial systems offer the most rapid, and cost‐effective means of antigen production (Pen & Meran, 1996). However, the bacterial cytoplasm does not provide the optimal milieu for the folding of target antigens of eukaryotic origin, and the antigens are usually expressed as insoluble aggregates. The downstream refolding process is capital‐intensive, inevitably requiring the solubilization of inclusion bodies in high concentrations of chaotropic agents such as GuCl or urea, followed by dilution, and further purification (Arakawa, 2010; Clark, 1998; Yamaguchi & Miyazaki, 2014). Solubilization based on chaotropic agents does not guarantee the retention of the conformation of the epitopes of the solubilized antigen. This compromises the quality of the Abs elicited by immunization. As an alternative to the chemical refolding process, the target antigen can be genetically fused to solubility‐enhancing proteins—for example, maltose‐binding protein (MBP), glutathione *S*‐transferase (GST), small ubiquitin‐like modifier (SUMO), or lysyl‐tRNA synthetase (LysRS)—and produced as soluble fusion proteins (Choi et al., 2008; Esposito & Chatterjee, 2006; Kapust & Waugh, 1999). Unfortunately, most fusion partners are of bacterial origin and therefore cannot be used as antigens owing to their immune dominance in the immunizing host. Fusion partners can be removed by cleavage at the linker, but this requires a site‐specific protease and further chromatographic purification steps. Insufficient cleavage is not unusual, even after prolonged treatment with a protease. This does not guarantee the purity of the antigen of interest, even after exhaustive purification, which ultimately compromises the quality of the antibodies after immunization.

Herein, we present a potentially universal platform for generating soluble recombinant antigens from bacterial hosts tailored to Abs production from a variety of animal hosts. A novel protein‐folding function of RNA has recently been recognized; it remarkably outperforms protein‐based molecular chaperones in promoting protein folding and solubility (Choi, Ryu, & Seong, 2009; Horowitz & Bardwell, 2016; J. M. Kim, Choi, & Seong, 2017; Kwon, Yu, Park, Lee, & Seong, 2018; Kwon, Kim, et al., 2018; Son, Choi, Han, & Seong, 2015). This unique function has been recognized both in natural cellular environments and the host–virus interface during viral infections and could be extended to the expression of soluble recombinant proteins (Yang et al., 2018). A convenient avenue for harnessing an RNA‐dependent chaperone (chaperna: chaperone + RNA) is to fuse the target antigen of interest with an RNA‐interacting domain (RID) as a docking tag. This enables interaction with cellular RNAs. Prominently, a small N‐terminal tRNA‐binding domain from LysRS, derived from the immunizing host animal, is selected as a transducer for the chaperna function of cellular tRNAs. The N‐terminal domain (~70 amino acids) of human LysRS is intrinsically disordered but switches from the unfolded to the alpha‐helical conformation during tRNA binding (Agou, Yang, Gesquiere, Waller, & Guittet, 1995). tRNA synthetases are known to interact with non‐cognate tRNAs with low affinity (Francin, Kaminska, Kerjan, & Mirande, 2002; Kwon, Yu, et al., 2018). This appended domain is present in all vertebrate LysRS enzymes and can therefore be derived from the immunized host animal and used to generate a soluble recombinant fusion antigen in bacterial hosts using the tRNA present in the bacterial cytoplasm as a chaperna. As a “self” protein, the appended RID is nonimmunogenic to the animal; therefore, the antibody (Ab) response predominantly targets the antigen without cross‐reactivity to the RID. Capitalizing on the novel RNA‐dependent chaperone function, the use of bacterially produced fusion proteins greatly simplifies the generation and screening of mono‐specific and monoclonal antibodies, which are used extensively in diagnostic and therapeutic applications.

Herein, we demonstrated the generality and practicality of the system using prominent examples of emerging and re‐emerging viral infections. Middle East respiratory syndrome (MERS) is a viral respiratory disease caused by the MERS corona virus (MERS‐CoV); there have been outbreaks of the disease in several countries including Saudi Arabia and the Republic of Korea (Al‐Omari, Rabaan, Salih, Al‐Tawfiq, & Memish, 2019; K. H. Kim, Tandi, Choi, Moon, & Kim, 2017). Moreover, there has been an unmet need for effective vaccines and diagnostic kits. As a proof‐of‐concept, in the present study we used the N‐terminal RID of a LysRS of murine origin to express the receptor‐binding domain (RBD) and HR2 domain of MERS‐CoV (Lu et al., 2013; Mou et al., 2013; Raj et al., 2013). The RID‐MERS RBD fusion protein was expressed in soluble form, purified, and used as an immunogen to screen hybridomas and identify mAbs with proven diagnostic potential when tested with MERS‐CoV‐infected patients. Influenza viruses circulate among the human population, and the continual emergence of genetically drifting strains necessitates annual vaccination. Moreover, the licensure of seasonal vaccines requires a potency test for vaccine lots each year (Houser & Subbarao, 2015; Marlet, Gaudy‐Graffin, Marc, Boennec, & Goudeau, 2018; Organization, 2017). Humans are still vulnerable to pandemics, as exemplified by the 2009 pdmH1N1 virus; therefore, improved vaccines and better therapeutic modalities are required (Newman et al., 2008; Shinde et al., 2009). In the present study, which is relevant to such global issues, we expressed the influenza virus hemagglutinin globular domain (HAgd) and the conserved stalk domain (the consensus hemagglutinin (cHA) stalk) using mouse RID (mRID) as a fusion tag to produce mAbs against the major influenza groups. Not only does the strategy produce mAbs that are suitable for potency assays for seasonal influenza vaccines, but the Fc‐effector function could also impact therapeutic and prophylactic applications. In principle, the present approach could be extended and tailored to any immunized animal, greatly increasing the repertoire of immunochemical applications.

## 2 MATERIALS AND METHODS

### 2.1 Expression and purification of proteins

All the plasmids were transformed into *Escherichia coli* strain BL21 Star (DE3) pLysS (Invitrogen, Carlsbad, CA). The cells were grown overnight at 37℃ in 50 ml of Luria broth (LB) media containing 1 mM ampicillin and chloramphenicol. The cells were inoculated into 500 ml LB media, and grown to an optical density of 0.8–1.0 (OD_600 nm_). Protein expression was induced by adding 1 mM isopropyl β‐d‐1‐thiogalactopyranoside and incubating overnight at 18℃. The cultured cells were harvested and were lysed in B‐PER (Thermo Fisher Scientific, Rockford, IL). All proteins with the 6 × ‐His tag were purified using a HisTrap HP column (GE Healthcare, Chicago, IL). The supernatant in a buffer comprising 50 mM Tris–HCl (pH 7.5), 300 mM NaCl, 10% glycerol, 2 mM 2‐mercaptoethanol, and 0.1% Tween‐20 was loaded onto a HisTrap HP column, and eluted with a linear gradient of imidazole in the same buffer. The physical properties were analyzed by Superdex 200 Increase 10/300 GL column (GE Healthcare).

### 2.2 RNA depletion by RNase A treatment

The cell culture and lysate experiments were carried out based on the protocols described in Section 2.2 of Yang et al. (2018). The *E. coli* total cell lysates (T) were centrifuged at 12,000 rpm for 10 min and separated into soluble (S) and pellet (P) fractions. The (S) fraction was divided into two vials (250 μg/ml): one vial was treated with RNase A (iNtRON Biotechnology, Seongnam, Republic of Korea) at 37℃ for 15 min, and the negative control was not treated with RNase A. The solution was further divided into soluble (SS) and precipitate (SP) fractions by centrifugation at 12,000 rpm for 15 min. All fractions were analyzed by sodium dodecyl sulfate polyacrylamide gel electrophoresis (SDS‐PAGE) (*n* = 3).

### 2.3 Co‐immunoprecipitation assay

MERS RBD (2 μg) and hDPP4 (2 μg) were diluted in 300 μl phosphate buffered saline (PBS) buffer and incubated overnight at 4℃ to induce binding between them. The anti‐hDPP4 antibody (Abnova, Taipei, Taiwan) was then added and the mixture was incubated overnight at 4℃. We added 10 μl of protein G agarose beads (50 μg/μl; Roche, Basel, Switzerland), and centrifuged the mixture at 12,000 × *g* for 30 s. The beads were washed three times with PBS, and the supernatant was discarded. Fresh cold PBS was added to the mixture, which was incubated on ice for 10 min. Finally, we obtained a pellet that comprised the beads and anti‐hDPP4 Ab bound to RBD and hDPP4. 2 × SDS was added to the mixture containing the pellets, which was boiled at 100℃ for 5 min. The boiled samples were electrophoresed on SDS‐PAGE and transferred to polyvinylidene difluoride membranes. For the western blot analysis, twofold diluted horseradish peroxidase‐conjugated anti‐6 × His tag monoclonal Ab (Thermo Fisher Scientific, Waltham, MA) was added, and the mixture was incubated for 1 hr at 37℃.

### 2.4 hDPP4 binding ELISA

We investigated the binding of RBD to hDPP4 protein, and the characteristics of 293T cells overexpressing hDPP4. A 96‐well immunoplate (Thermo Fisher Scientific, Waltham, MA) was coated with 5 μg/ml hDPP4 protein (Abcam, Cambridge, UK) or 2 × 10^5^ 293T cells overexpressing hDPP4 (Invitrogen, Life Technologies) (Kim et al., 2016), and incubated overnight at 4℃ (100 μl/well). The plates were washed three times with phosphate‐buffered saline with Tween‐20 (PBST). We added 200 μl of the blocking buffer (5% skim milk in PBST) to each well, and stored the plates at room temperature for 1 hr. After removing the buffer, 100 μl of diluted 6 × ‐His tagged mRID‐RBD (5 μg/ml) from *E. coli*, or 293T cell derived 6 × ‐His tagged RBD (5 μg/ml, MERS‐RBD‐005P; eEnzyme) was added, and incubated at 37℃ for 2 hr. Subsequently, we added anti‐6 × His tag Ab (diluted by 1/1,000; Qiagen, Hilden), and incubated the mixture at 37℃ for 1 hr. Then, anti‐mouse IgG Ab (diluted by 1/5,000; Sigma Aldrich, St Louis, MO) was added and incubated the mixture at 37℃ for 1 hr. Each of the steps described above was followed by washing with PBST. Finally, we added a 3,3',5,5'‐Tetramethylbenzidine (TMB) substrate solution to each well (100 μl/well), and stopped the enzymatic reactions with 50 μl of 2 N H_2_SO_4_. The optical density at 450 nm was measured using an ELISA plate reader (FLUOstar Optima; BMG Labtech, Ortenberg, Germany).

### 2.5 Ethics statement

Animals were used to generate polyclonal serum for the experiments. We immunized 6‐week‐old female BALB/c mice with the MERS‐CoV antigens. The experiments were conducted under the guidelines provided by the Ministry of Food and Drug Safety (MFDS) of the Republic of Korea. All protocols related to the study were reviewed and approved by the Yonsei University Institutional Animal Care and Use Committee (IACUC; permit number: IACUC‐201606‐445‐01). The sera from seven patients infected with MERS‐CoV were approved by the institutions of Chungnam National University Hospital (IRB, 2015‐08‐160 029) and Seoul National University Hospital (IRB, 1509‐103‐705). The study was conducted in accordance with the ethical standards set out in the 1964 Declaration of Helsinki and all subsequent revisions. Informed consent was obtained from all the patients who participated in the study.

### 2.6 Immunization of mice and serum collection

We obtained serum from 6‐week‐old female BALB/c mice immunized with mRID‐RBD or HR2 (20 μg/mouse) adjuvanted with alum (Thermo Fisher Scientific, Waltham, MA). PBS and mRID with alum were used as negative controls. Each immunized mouse was boosted twice by intramuscular (i.m) injections once every 2 weeks. The mice bled from the eyes, providing us with blood samples; the immune sera were harvested from the supernatants.

### 2.7 ELISA for serodiagnosis

We used an ELISA to determine whether the pAbs obtained from the immunized mice or from the infected human serum could bind to RBD and HR2. We coated RBDs produced from 293T cells (MERS‐RBD‐005P; eEnzyme; positive control), and HR2 and mRID (negative control) onto 96‐well Nunc immunoplates (Thermo Fisher Scientific, Waltham, MA), and incubated them overnight at 4℃. A blocking buffer (5% skim milk in PBST) was added and the mixture was incubated for 2 hr at 37℃. Starting from 10 to 0.1 μg, we added pAbs obtained from inactivated MERS‐CoV‐immunized mice and from the infected patient serum (diluted by 1:50; 100 μl/well), and incubated the mixtures for 2 hr at 37℃. We then added secondary Ab (diluted by 1/3,000; goat anti‐human IgG Ab, KPL) in 2% skim milk, and incubated the mixture for 1 hr at 37℃. TMB substrate was added (50 μl/well), and the mixture was incubated for 10 min. The reactions were stopped by adding 50 μl of 2 N H_2_SO_4_, and the optical densities at 450 nm were measured using an ELISA plate reader.

### 2.8 Production of monoclonal antibodies

MAbs against each fusion proteins were generated from murine cell fusion/hybridoma by ATGen (Seongnam, Korea). Six‐week‐old BALB/c mice were immunized with purified mRID‐RBD, mRID‐cHA stalk (IAV) and mRID‐cHA stalk (IBV), respectively, via intraperitoneal injection (IP). The mice were boost‐immunized twice with the same fusion proteins. The mAbs were generated using hybridoma fusion technology (Hendriksen & de Leeuw, 1998; Institute of Laboratory Animal Resources U.S. Committee on Methods of Producing Monoclonal Antibodies., & National Research Council U.S., 1999). Hybridoma clones were selected by the ELISA response to mRID‐RBD and mRID‐cHA stalk from IAV and IBV, and selected against mRID (1 μg/ml). The specificity of the mAbs against influenza HA was confirmed by ELISA assay. We purified mAbs from the positive clones using Protein G (GE Healthcare, Chicago, IL).

### 2.9 Indirect ELISA

We produced influenza H5gd (hemagglutinin globular domain from influenza virus H5N1) and stalk antigens by fusion to mRID, and pretreated them in various buffers according to a previously reported method (38). Egg‐derived influenza vaccine antigens—H1N1 (A/Singapore/GP1908/2015 IVR‐180), Yamagata lineage (B/Phuket/3073/2013), and Victoria lineage (B/Maryland/15/2016 NYMC BX‐69A)—were supplied by Green Cross Pharma, Republic of Korea. We coated twofold serially diluted HA antigens (1.35 μg/ml) on 96‐well microplates (NUNC, Roskilde, Denmark), and incubated them overnight at 4℃. The plates were washed three times with PBST following each step. We blocked the wells with 5% skim milk in PBST for 1 hr at 37℃. After washing with PBST, IAV specific‐mAb #5 (1 ug/ml) and IBV specific‐mAb #3 (1 ug/ml) were serially diluted by two‐fold in TBST (50 mM Tris‐Cl (pH 7.4) with 0.05% Tween‐20). 50ul of diluted mAb were added to each wells, and incubated for 2 hr at 37°C. ELISA were carried out based on the protocols described in hDPP4 binding ELISA.

### 2.10 Ab‐dependent cellular cytotoxicity assay

MDCK cells were cultured according to established protocols for use in the Ab‐dependent cellular cytotoxicity (ADCC) Reporter Bioassay. ADCC activity of mAb (IAV, IBV) was tested using H1N1 (A/Puerto Rico/8/34) and B/Yamagata/16/88 viruses. First, MDCK cell (each well) was combined with H1N1 and B/Yamagata viruses (1MOI), mixed well, and infected for 45min at 37℃. After washing with PBS, the plates were incubated at 37℃ overnight in MEM (serum‐free, 40μl Trypsin/10ml MEM). After washing with PBS, IAV specific‐mAb #5 (1mg/ml) and IBV specific‐mAb #3 (1mg/ml) were serially two‐fold diluted in TBST (50mM Tris‐Cl, pH 7.4; 0.05% Tween‐20), added at 50μl to the wells, and incubated for 2hr at 37°C. And put the prepared mFcγRIV Effector Cells in 25μl/well and incubated for 6hr. ADCC activity was measured with Promega mFcγRIV ADCC Reporter Bioassay (Promega; M1402). The luminescence was measured with a FLUOstar OPTIMA microplate reader.

## 3 RESULTS

### 3.1 Development of target proteins

**Figure 1:**
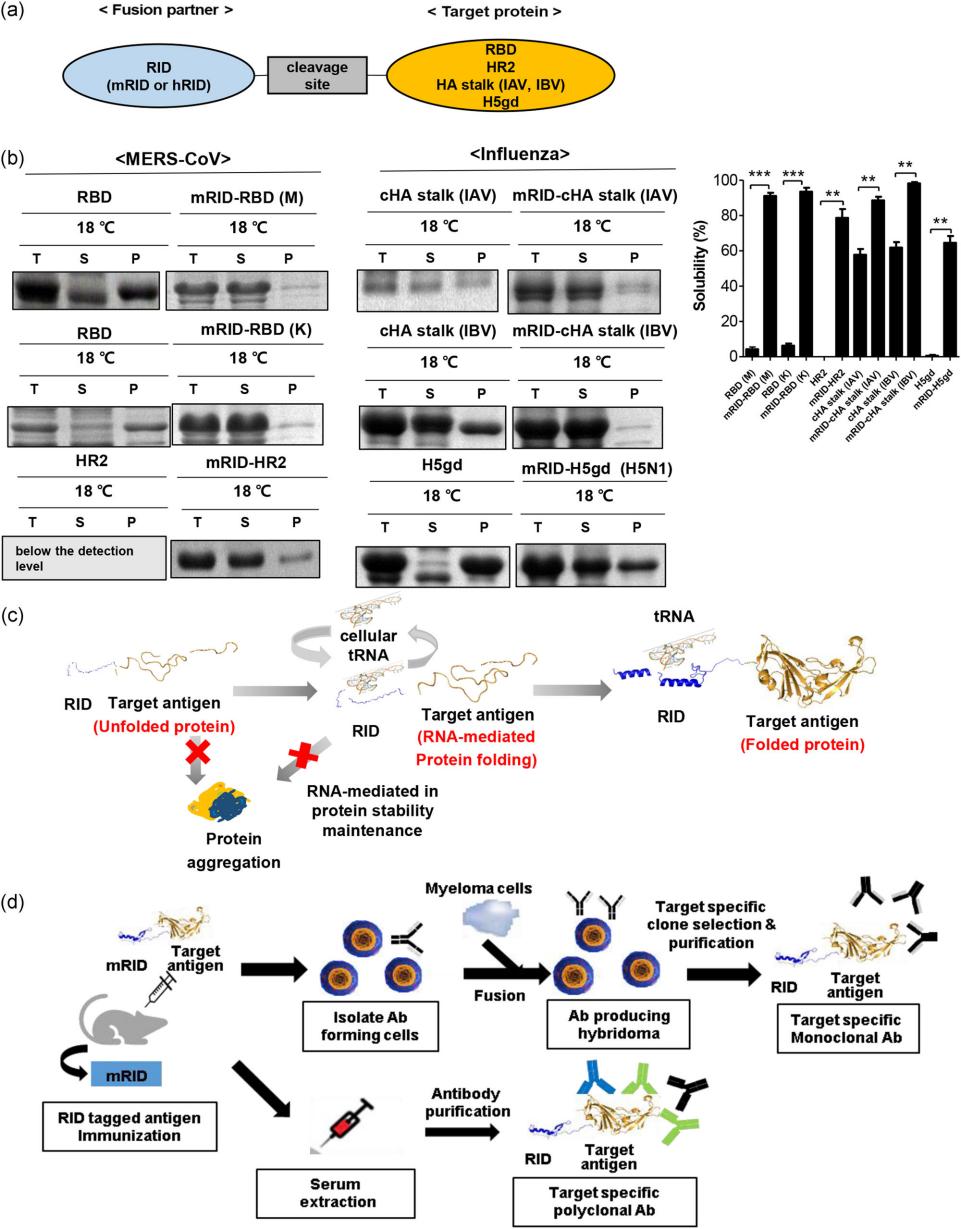
Schematic illustration and expression of soluble antigens in *Escherichia coli*. (a) Schematic structure of pGE‐mRID‐fused RBD and HR2 linked by a TEV protease cleavage site. (b) The solubilities of the mRID‐fused RBD, the HR2 domain from the MERS CoV spike protein, the cHA stalk (IAV), the cHA stalk (IBV), and the H5gd from influenza hemagglutinin (HA) were analyzed by sodium dodecyl sulfate–polyacrylamide gel electrophoresis. The solubilities (%) were estimated by scanning the protein band of interest with a densitometer, and the data are presented as mean ± *SD* (*n* = 3). **p < .01, ***p < .001. (c) Schematic illustration of the RNA‐mediated protein folding model using an RID as a fusion tag. RNA binds to the RID and exerts chaperone function toward productive folding while minimizing off‐pathway aggregation. The RID and the target protein are represented by blue and brown, respectively. (d) Schematic diagram of the antibody production method using the RID fusion tag. Ab, antibody; CoV, corona virus; HA, hemagglutinin; hRID, human RID; IAV, influenza A virus; K, Korean strain; M, Middle East strain; MERS, Middle East respiratory syndrome; mRID, mouse RID; P, pellet; RBD, receptor‐binding domain; RID, RNA‐interacting domain; S, supernatant; SDS‐PAGE, sodium dodecyl sulfate polyacrylamide gel electrophoresis; T, total lysate; TEV, tobacco etch Virus; tRNA, transfer RNA [Color figure can be viewed at wileyonlinelibrary.com]

The spike glycoprotein (S) of MERS‐CoV, which is displayed on the viral membrane surface, is responsible for the initiation of infection and subsequent pathogenesis (Lu et al., 2013; Raj et al., 2013). Consequently, based on the antigenicity prediction, we selected the RBD (367–606 amino acids) and the HR2 domain of the S protein (1246–1295 amino acids) as target antigens. The cHA stalk of influenza HA were generated based on the consensus sequences of group 1 influenza A viruses (IAVs), as previously described (Figure 1a; Chae, Kim, Hwang, & Seong,  2019). The globular domain (gd) of HA was selected from influenza virus H5N1. The RBD, cHA stalk (IAV, IBV), and H5gd proteins were all produced predominantly as insoluble aggregates, and the HR2 levels were below the detection level (Figure 1b). Thus, the target antigens were not amenable to soluble expression by conventional direct expression. When fused to lysyl‐tRNA synthetase (LysRS)‐derived RID, however, their solubilities greatly increased (Figure 1b); mRID fusion markedly increased the solubility of the cHA stalk of IAV (~30.8%), the cHA stalk of IBV (~36.4%), and H5gd (~64.5%), compared to direct expression without fusion. Likewise, the fusion of mRID greatly increased the solubility of the recombinant MERS‐CoV antigens, with results of 91.1% versus 4.3% for the Middle East strain, and 91.1% versus 6.0% for the Korea strain (Figure 1b). Furthermore, 78.6% of the mRID‐HR2 (the fusion form) was soluble, whereas HR2 (the direct form without fusion) was expressed predominantly in the insoluble form (Figure 1b). These results are consistent with and further extend previous observations of chaperna's function, mediated by RNA interactions (Figure 1c; Kwon, Yu, et al., 2018; Yang et al., 2018). The increase in the solubility of proteins at low temperature is probably due to the slowing down of translation, which provides a kinetically favorable environment for the folding of nascent proteins. All of the proteins were efficiently purified by one‐step affinity chromatography, suggesting that fusion with RID effectively triggers the folding and solubility of MERS‐CoV antigens.

RIDs occur as N‐terminal‐appended domains of LysRS in various animal hosts. In the present study, we compared RIDs from three different sources: mice (mRID), humans (hRID), and chickens (cRID; Figure S1). RIDs themselves are highly soluble, regardless of their origin, which is a requirement of successful solubility enhancer function. We chose mRID as a fusion tag; mouse immunization is the most popular method of polyclonal and monoclonal Ab generation, and the “self” mRID domain is not immunogenic. Therefore, unwanted, nonspecific immune responses to fusion tags during murine mAb generation are minimized (Figure 1d). Furthermore, the source of the RID can be tailored to the immunized host animal for specific Ab generation.

### 3.2 Influence of RNA on protein stability in vitro

**Figure 2:**
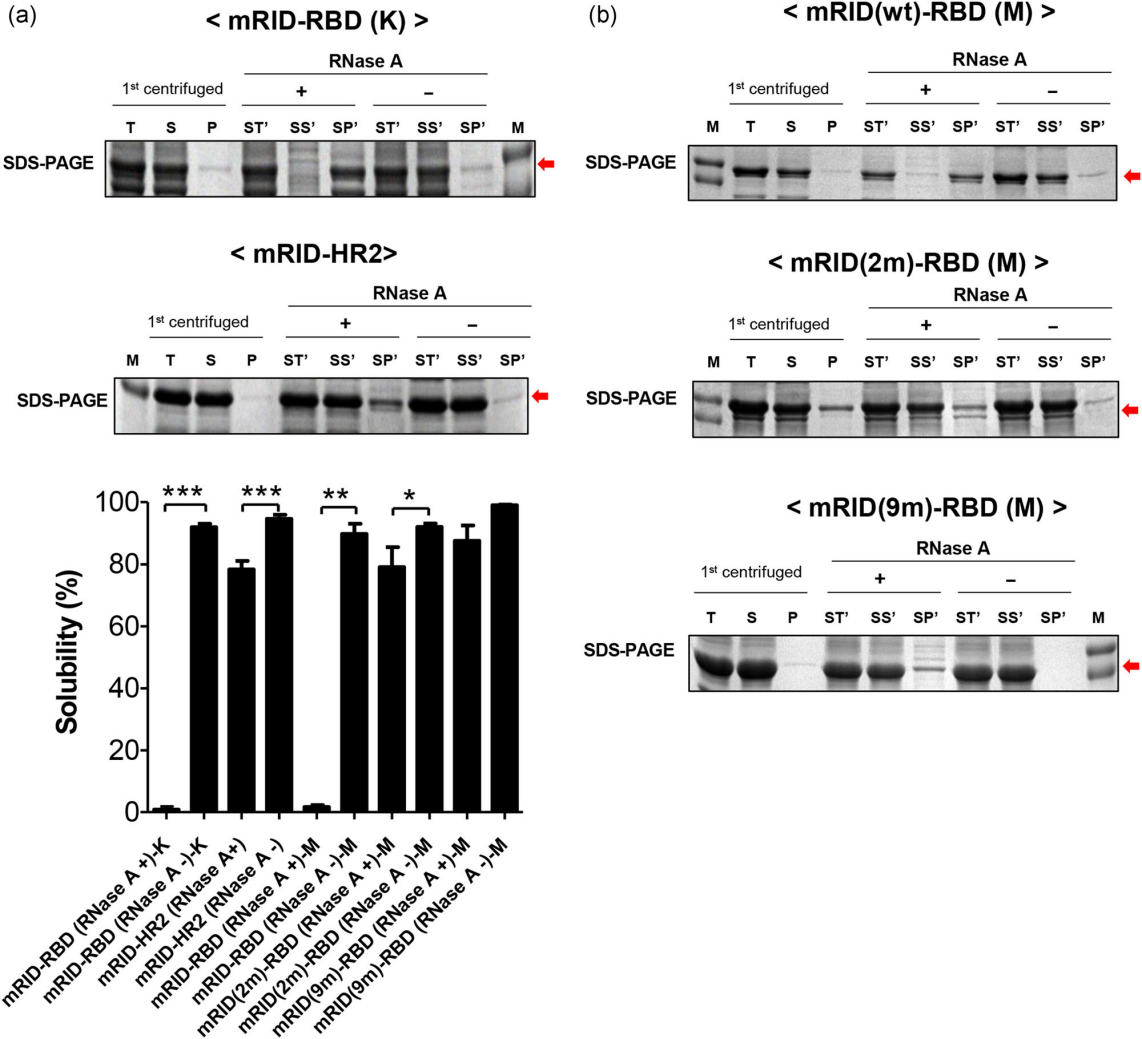
Effect of RNA binding on protein solubility. (a) SDS‐PAGE analysis of mRID‐fused proteins RBD and HR2) with (+) and without (−) RNase A treatment. (b) Effect of RNA depletion on the solubility of mRID‐RBD as analyzed by SDS‐PAGE (wt =  wild type; 2 m =  double mutant; 9 m =  nine‐fold mutant) (Table S1). The cells were lysed with B‐PER II and incubated with RNase A at 37℃. The total fraction (ST′) was separated into soluble (SS′) and insoluble (SP′) fractions by centrifugation. All proteins were expressed at 18℃. M, T, S, and P represent the molecular weight marker, the total lysates, the soluble fraction, and the insoluble fraction, respectively. Target proteins are indicated by red arrows. All data are presented as mean ± *SD* (*n* = 3). K, Korea strain; M, Middle East strain; mRID, mouse RNA‐interacting domain; RBD, receptor‐binding domain; SDS‐PAGE, sodium dodecyl sulfate polyacrylamide gel electrophoresis. *p* Values were determined by two‐tailed Student's tests (**p < .01, ***p < .001) [Color figure can be viewed at wileyonlinelibrary.com]

Recent studies on protein folding have provided insight into a novel function of RNA molecules as chaperones that assist the folding of nascent polypeptides and prevent misfolding into nonfunctional aggregates (J. M. Kim et al., 2017; Kwon, Kim, et al., 2018). To verify the effect of RNAs on the physical stability of RID‐fused proteins, we carried out cell lysis using a mild buffer (B‐PER II) to minimize the dissociation of bound RNAs. The total cell lysates (T) were divided into a soluble fraction (S) and a pellet fraction (P) by centrifugation. The soluble fraction (S) was then treated with RNase A and centrifuged into a further two fractions: soluble (SS) and precipitate (SP) fractions. mRID‐RBD, which initially retained high solubility, was rendered completely insoluble by RNase A treatment (Figure 2a). With mRID‐HR2, we also noted an increase in the level of insoluble precipitate upon RNA depletion (RNase A + ) compared to the level in the RNase A‐ control (Figure 2a). The results demonstrate that RNA can protect aggregation‐prone proteins (mRID‐RBD and mRID‐HR2, in the present study) from misfolding and precipitation, which is reminiscent of the function of molecular chaperones in proteostasis (Herschlag, 1995; Moll, Leitsch, Steinhauser, & Bläsi, 2003). The retention of the solubility of RNA‐binding mutants regardless of the presence of RNAs may result from the highly disordered nature of mRID, which is an intrinsically disordered protein (IDP). This extends the findings of previous researchers who attempted to increase the solubility of target proteins by linking them to IDPs (Kwon, Yu, et al., 2018; Santner et al., 2012; Yang et al., 2018). The RNA dependence of solubility observed in wild‐type (wt) proteins may be associated with the conformational transition from disorder into alpha‐helical structure upon RNA binding.

### 3.3 Influence of RNA on RID folding

The physicochemical properties of antigens were analyzed by Ni‐NTA column and SEC. The soluble fraction in Figure 2 was subjected to Ni‐NTA column (Figure S3). With RNA‐binding mutants (e.g., mRID(2 m)‐RBD and mRID(9 m)‐RBD), significant portion of the proteins failed to bind the column, enriching the flow‐through fractions (frs 1 & 2). The failure to bind to Ni‐NTA is probably due to the masking of the His‐tag due to nonfunctional soluble aggregations. In contrast, wt RID‐RBD mostly binds to the affinity column and eluted on imidazole gradient. Next, the pooled fractions of the Ni‐affinity chromatography proteins were further subjected to SEC (Figure S4). The elution profile of mRID(wt)‐RBD obtained by SEC was fractionated into oligomeric (fractions #2) and trimeric (fractions #8) forms, whereas mRID(9 m)‐RBD was predominantly present as high‐molecular‐weight oligomeric forms (fractions #2). The trimeric status of the assembly is also apparent in mRID‐HR2 (fractions #12) by SEC analysis (Figure S4). Thus, the assembly repertoire in favor of (biologically relevant) trimer formation is triggered by RNA‐binding. Defective RNA‐binding results in irregular assembly into soluble aggregates. It should be noted that each of the RNA‐binding mutants was expressed in its soluble and purified form (Figure S2), and remained soluble even after RNase A treatment (Figure 2b), in clear contrast to the behavior of the wild‐type following similar treatment (Figure 2a). Overall, especially considering the defective binding to the affinity column (Figure S3), the RNA‐binding mutants were expressed as soluble aggregates, but had biologically irrelevant conformations. Therefore, RNA interaction is crucial for the folding of monomers and their subsequent assembly into biologically/immunologically relevant oligomeric (here, in this case, trimeric) conformations (Figure 1c).

**Figure 3:**
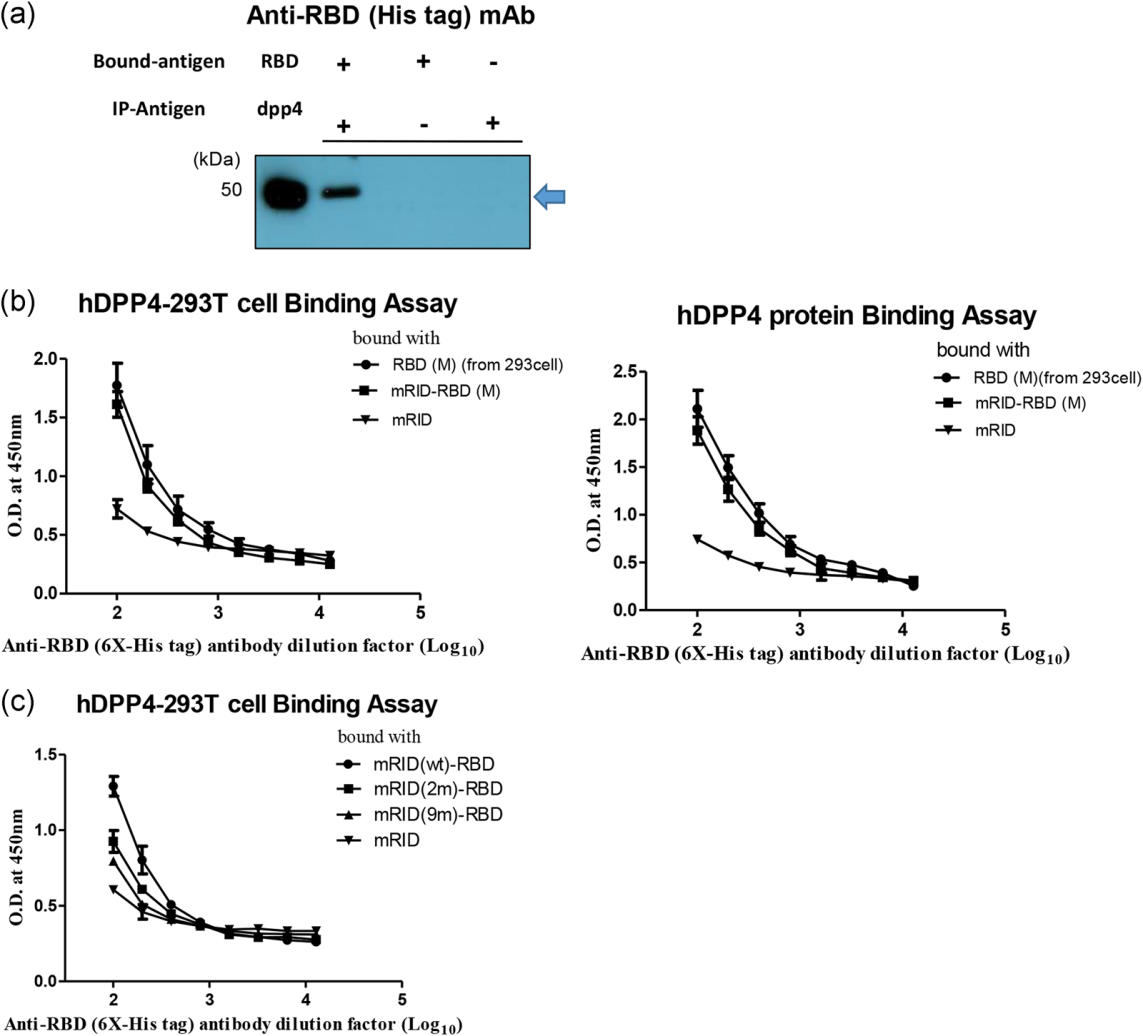
Binding between RBD and hDPP4. (a) Co‐immunoprecipitation analysis of MERS RBD (2 μg, *Escherichia coli*) binding to hDPP4 protein (2 μg, 293 cell) using protein G (50 μg/μl) and hDPP4 specific antibody. RBD protein (his tagged) was detected by western blot analysis using anti‐6 × ‐His tag monoclonal antibody. (b) Dose‐dependent binding of His‐tagged RBD (5 μg/ml) from *E. coli* and 293T cell (eEnzyme), respectively, with hDPP4 (recombinant or ectopically displayed in 293T cells) as coating antigen determined by ELISA. RBD(M) (derived from *E. coli*) was compared with mRID (the negative control) and RBD from 293T cells (the positive control). Three independent experiments were performed (*n* = 3). (c) Binding ELISA of mRID (wild‐type (wt), 2 m, 9 m)‐RBD and 293T cells overexpressing hDPP4. The ELISA data were obtained in duplicate. The antibodies were twofold serially diluted from 1:100. All data are presented as mean ± *SD*. ELISA, enzyme‐linked immunosorbent assay; K, Korean strain; M, Middle East strain; MERS, Middle East respiratory syndrome; mRID, mouse RNA‐interacting domain; RBD, receptor‐binding domain [Color figure can be viewed at wileyonlinelibrary.com]

The RBD of MERS‐CoV is responsible for binding to the cellular receptor hDDP4 to initiate infection (Lu et al., 2013; Mou et al., 2013; Raj et al., 2013). Co‐immunoprecipitation confirmed that the mRID‐RBD protein produced by the *E. coli* bound to the DPP4 receptor (Figure 3a). We then performed a receptor binding assay. The ELISA was performed using recombinant hDPP4 and 293T cells overexpressing hDPP4 as coating antigens (Figure 3b). The results showed that mRID‐RBD bound to the recombinant hDDP4 as efficiently as to the 293T cells overexpressing hDPP4. Notably, the binding efficiency decreased markedly with the RNA‐binding mutants mRID(2 m)‐RBD and mRID(9 m)‐RBD. The lack of binding was probably due to the formation of soluble aggregates (Figures S3 and S4). The results suggest that the folding of RBD into a biologically relevant conformation is indeed mediated by RNA interaction. The negative control mRID failed to bind, confirming that the binding is specific to RBD–hDPP4 interaction. In conclusion, RNA interaction plays a crucial role in the folding of MERS‐CoV RBD into a biologically functional conformation.

### 3.4 Serological diagnosis of MERS‐CoV‐infected patients

**Figure 4:**
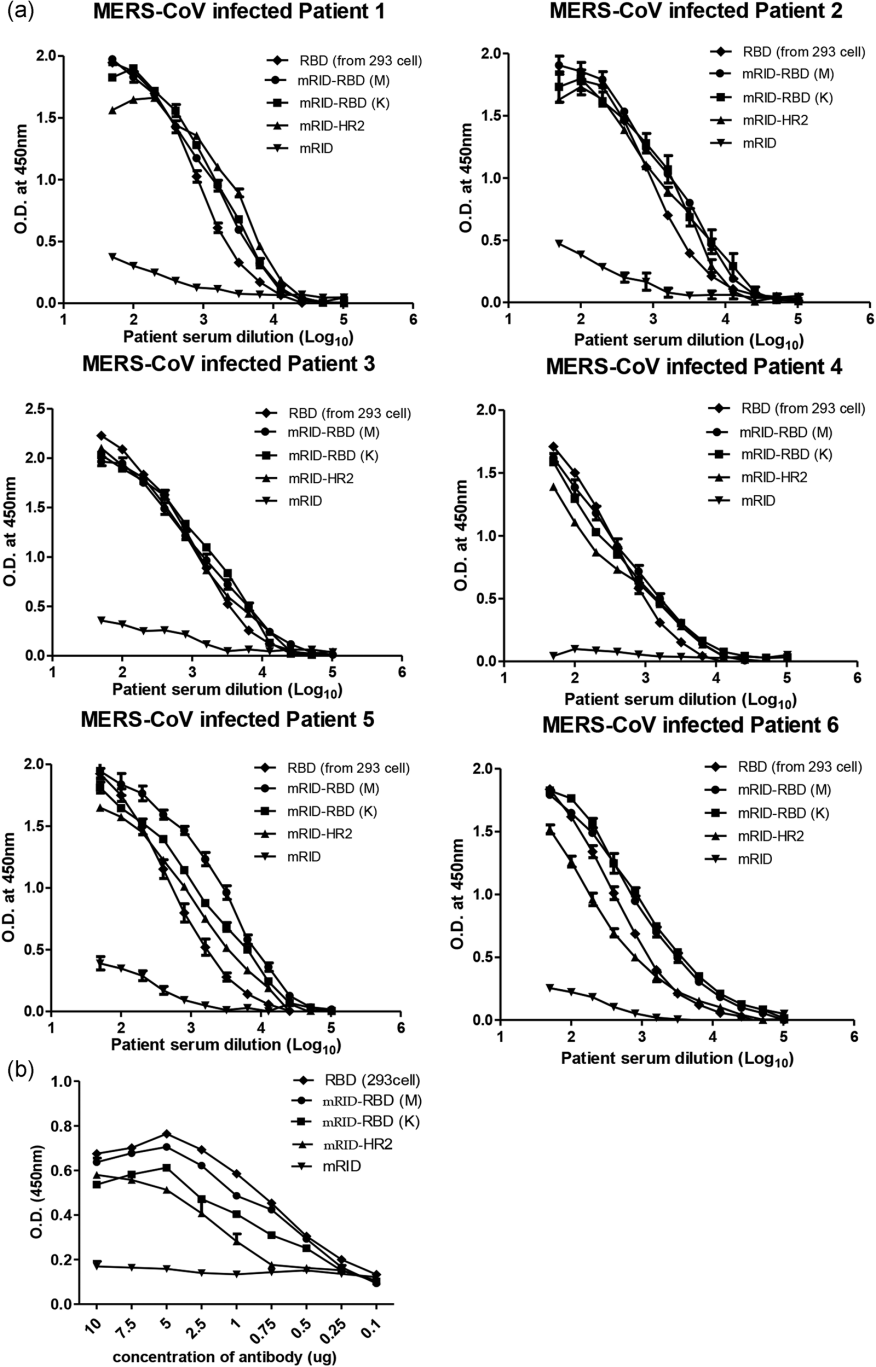
Serodiagnosis of MERS‐CoV infection in humans using RBD and HR2 as diagnostic antigens. (a) MERS‐CoV‐specific antibodies in the infected patients sera were detected by an indirect ELISA. ELISA plates were coated with viral antigen (RBD, mRID‐RBD(M), mRID‐RBD(K), mRID‐HR2) and detected with patients sera. The sera from six human patients were twofold serially diluted from 1:50. (b) ELISA of *Escherichia coli*‐derived RBD using polyclonal antibodies (mouse) obtained by immunization with inactivated MERS‐CoV. The polyclonal antibodies were twofold serially diluted from 10 μg. RBD (from Middle East and Korea strains) and HR2 proteins were coated on a microplate. mRID and RBD (from 293T cells) were used as negative and positive controls, respectively. All data are presented as mean ± *SD* (*n* = 3). ELISA, enzyme‐linked immunosorbent assay; MERS CoV, Middle East respiratory syndrome corona virus; mRID, mouse RNA‐interacting domain; RBD, receptor‐binding domain

Previous studies have shown that antibodies against spike proteins are induced upon MERS‐CoV infection (Jiang et al., 2014; Song et al., 2013; Zhang, Jiang, & Du, 2014). Therefore, both the RBD and the HR2 domain derived from the spike protein could be used as sero‐diagnostic antigens for MERS‐CoV infection. Thus, mRID‐RBD from both the Korea (K) and Middle East (M) strains, and mRID‐HR2 were used as coating antigens for MERS‐CoV serodiagnosis, using RBD (from 293T cells and from *E. coli*) and mRID (produced by *E. coli*) as positive and negative controls, respectively. ELISA was performed using sera from six patients infected with MERS‐CoV (Chungnam National University Hospital, Republic of Korea). All the sera from the MERS‐CoV‐infected patients reacted with mRID‐RBD (either of Korean or Middle Eastern isolates) and mRID‐HR2 in the ELISA format. It is worth noting that the binding abilities of the *E. coli*‐derived RBD and HR2 were similar to the binding ability of the RBD produced from the 293T cells (the positive control; Figure 4a). In parallel, we performed an ELISA using the sera from inactivated MERS‐CoV‐immunized mice (Joong Kyeom Co., Republic of Korea). The mRID‐RBD and mRID‐HR2 bound efficiently with the mouse sera (Figure 4b). The carrier protein mRID only failed to react with the sera from either infected patients or immunized mice, confirming that RBD and HR2 antigens are suitable for the diagnosis MERS‐CoV infection.

### 3.5 Target specificity of polyclonal antibodies

**Figure 5:**
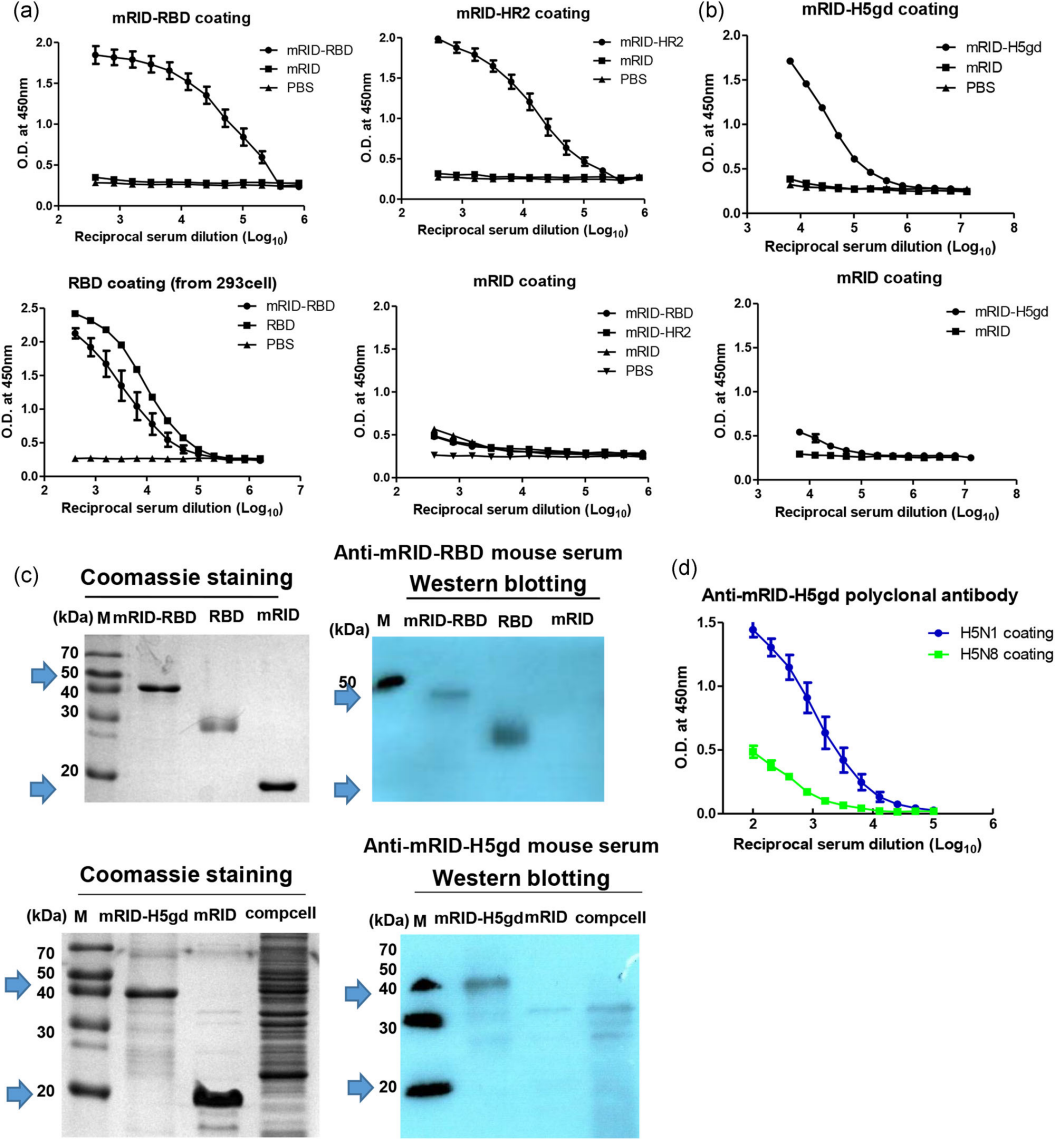
ELISA and western blot analysis of recombinant viral antigens. (a) ELISA analysis of IgG titers in mouse serum immunized with mRID‐RBD and mRID‐HR2. mRID‐RBD, mRID‐HR2, mRID (the negative control), and RBD from overexpressing 293T cells (the positive control) were used as coating antigens. (b) ELISA titers of anti‐mRID‐H5gd immune sera. mRID‐H5gd and mRID were used as coating antigens. All mouse sera were twofold serially diluted (MERS‐CoV serum and influenza serum starting from 1:200 and 1:6,400, respectively). All data are presented as mean ± *SD* (*n* = 5). (c) RBD‐ and H5gd‐specific polyclonal antibodies were detected by western blot analysis using mRID‐RBD, RBD (from 293T cells; upper panel), and influenza H5gd (lower panel). The target proteins are represented by blue arrows. All proteins were purified using a single Ni–NTA affinity column. (d) Detection of H5N1‐specific antibodies in mouse sera. Egg‐derived HA antigens from H5N1 and H5N8 strains (Green Cross Pharma, Republic of Korea) were used as coating antigens. The sera were serially diluted from 1:100. ELISA, enzyme‐linked immunosorbent assay; IgG, immunoglobulin G; MERS CoV, Middle East respiratory syndrome corona virus; mRID, mouse RNA‐interacting domain; RBD, receptor‐binding domain [Color figure can be viewed at wileyonlinelibrary.com]

To verify the target specificity, we tested the sera from mRID‐RBD and mRID‐HR2‐immunized mice with various antigens—that is mRID, mRID‐RBD, HR2, and RBD from 293T cells (sera concentrations starting from 1/200 dilution)—by ELISA (Figure 5a). The RBD‐ and HR2‐specific Ab titers of the mRID‐immunized sera (the negative controls) and the PBS control mice were extremely low even at the highest concentration tested, but the RBD‐ and HR2‐specific Ab titers of the RBD‐ and HR2‐immunized sera were high (relative titer > 1,000‐fold). Basically, the sera were specific to MERS antigen and did not cross‐react with mRID (the fusion tag). The ELISA response to *E. coli*‐derived mRID‐RBD was as pronounced as the response to mammalian cell‐produced RBD. In parallel, we also used an ELISA to verify that polyclonal antibodies from mRID‐H5gd‐immunized mice had much higher reactivity to H5gd than to mRID (Figure 5b). The western blot analysis further verified specific target antigen binding without cross‐reactivity to mRID (Figure 5c). The results showed that the immune response was elicited predominantly to the desired target antigen rather than to the fusion partner of self‐origin. Finally, the ELISA showed that polyclonal antibodies specifically detected influenza H5N1 without cross‐reactivity with H5N8 (Figure 5d). The specificity observed even within the same H5 subtypes is probably an outcome of the immunological quality of the mRID fusion proteins as antigens. The results suggest that the RID derived from a murine host could be used as a fusion partner for the folding of target antigens into immunologically relevant conformations, without compromising binding specificity. In conclusion, the “self” RID derived from the immunized host is not immunogenic, and yet can be used to transduce RNA‐dependent folding of a target antigen into a soluble and immunologically relevant conformation.

### 3.6 Development of specific mAbs against MERS‐CoV RBD and influenza HA

**Figure 6:**
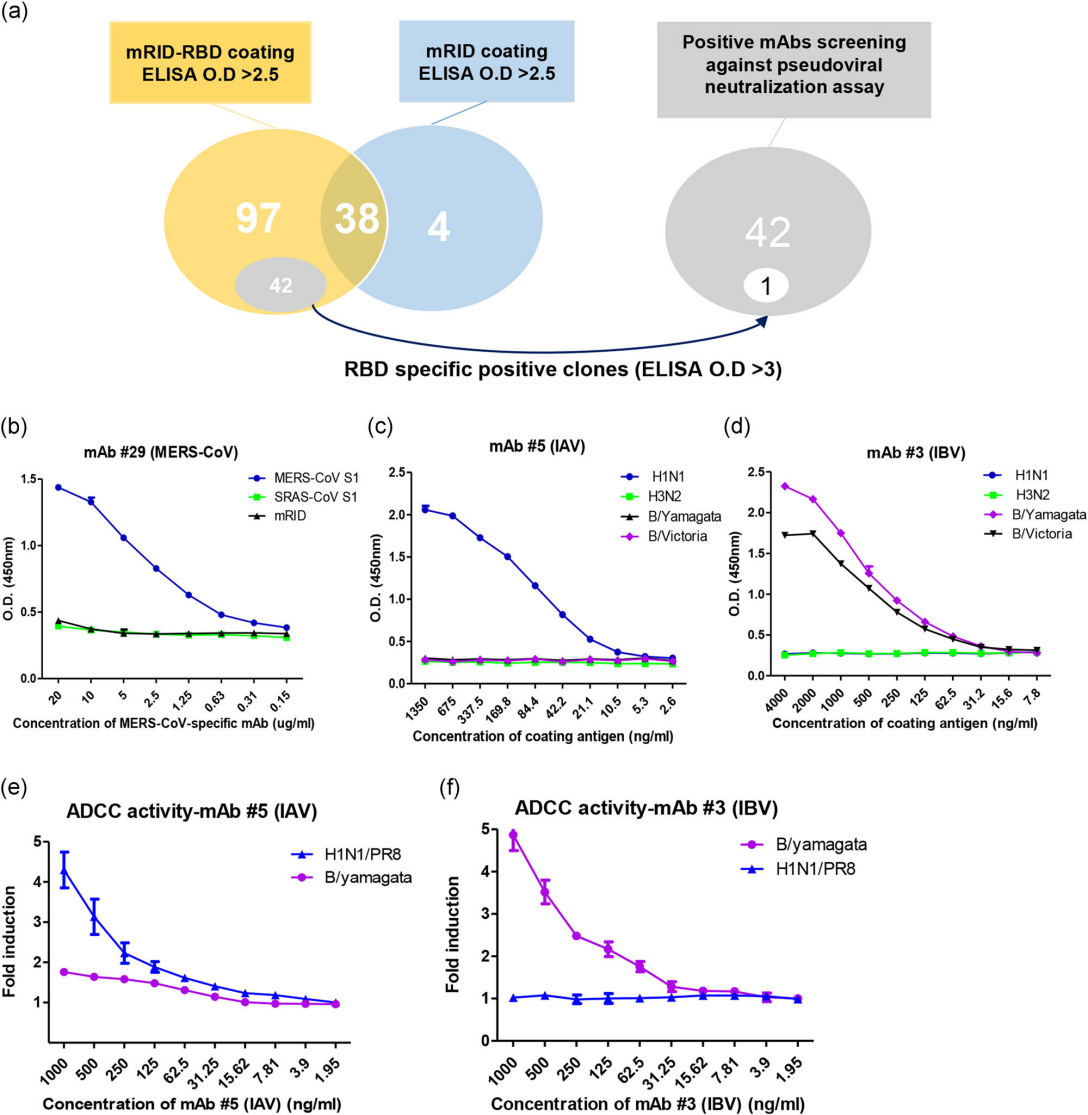
Screening and validation of the specificities of monoclonal antibodies against MERS‐CoV RBD, cHA‐stalk (IAV) and cHA‐stalk (IBV) using recombinant mRID‐fusion proteins as coating antigens. (a) Venn diagram of specific mAbs against MERS‐CoV RBD. BALB/c mice were immunized with mRID‐RBD to generate MERS‐CoV‐specific mAb. Positive hybridoma clone were screened by ELISA using mRID‐RBD (ELISA O.D > 3) (positive selection) and mRID (negative selection) as coating antigens. (b) Binding specificity of mAb for MERS‐CoV spike protein. S1 protein from SARS‐CoV and mRID were used as the negative controls. MERS‐CoV‐specific mAb was serially diluted from 20 μg/ml stock. Validation of group‐specific mAbs against IAV (c) and IBV HAs (d) (egg‐derived influenza HAs from H1N1 (Group 1), H3N2 (Group 2), B/Yamagata‐like, and Victoria‐like) by ELISA analysis. The HA proteins were twofold serially diluted from 1.35 and 4 μg/ml. Analysis of in‐vitro ADCC activities of mAb (IAV) (e) and mAb (IBV) (f). IAV specific‐mAb #5 and IBV specific‐mAb (1 μg/ml) were serially diluted by two‐fold. All data are presented as mean ± *SD* from triplicate samples. H1N1, B/Yamagata viruses were used to infect cells. ADCC, Ab‐dependent cellular cytotoxicity; ELISA, enzyme‐linked immunosorbent assay; HA, hemagglutinin; IAV, influenza A virus; IBV, influenza B virus; mAb, mouse Ab, antibody; MERS CoV, Middle East respiratory syndrome corona virus; RID, mouse RNA‐interacting domain; RBD, receptor‐binding domain [Color figure can be viewed at wileyonlinelibrary.com]

Following one‐step Ni‐affinity chromatography, we used mRID‐RBD to generate mAbs using hybridoma technology. Initially, we screened a total of 139 independent clones for a positive ELISA response to MERS‐CoV (Figure 6a). We found 97 clones specific to mRID fused MERS‐CoV RBD. Among 97 positive clones, we selected 42 RBD specific clones (ELISA O.D > 3) that specifically bind to RBD (positive selection), but not to mRID (negative selection; Figure 6a). Much small number (total 4) of clones appeared to be mRID‐specific, resulting in the Ab repertoire of 96% (97/97 + 4) preponderance toward the target antigen RBD. A total of 38 other clones reacted positively to both the mRID‐RBD and mRID preparations. They were probably reacting to contaminating proteins of *E. coli* origin present in the one‐step affinity‐purified protein preparations (Figure 5c). Based on the total clones examined, the screening efficiency was 69.8% (97/139; Table 1). One of the selected 42 RBD specific positive clones responded positively to MERS‐CoV pseudovirus neutralization assay (data not shown). MAb#29 (positive Selected mAb) was identified to specifically bind to MERS‐CoV spike protein only without cross reactivity to mRID (the fusion tag) or other coronavirus‐derived spike protein (SARS‐CoV; Figures 6b and S5).

**Table 1:** Screening of specific mAb clones against MERS CoV cHA stalk (IAV and IBV)

Immunized antigen	Sequences of target antigen (strain)	Number of positive clones using ELISA (coating antigen)			Efficiency of positive mAb screening
		mRID‐RBD	mRID	Both	Specific mAbs against target antigen (including all clones)
mRID‐RBD (MERS‐CoV)	Human betacoronavirus 2c EMC/2012	97	4	38	96% (69.8%)
mRID‐cHA stalk (IAV)	High frequency fragments of subtype H1, H2, H5, H9	15	0	ND	100%
mRID‐cHA stalk (IBV)	Consensus HA stalk sequences of 6 IBV	13	0	1	100% (92.85%)

We conducted additional experiments targeting influenza viral protein. cHA stalk represents the consensus sequence of the conserved stalk domain influenza HA protein. The HA stalk draws attention as potential target for universal influenza vaccine (Jang & Seong, 2019), and an ELISA‐based influenza vaccine potency assay (Chae, Kim, Kim, et al., 2019). Thus, mRID‐cHA stalk (IAV) was constructed based on the HA sequence of influenza A group1 viruses (H1, H2, H5 and H9), and mRID‐cHA stalk (IBV) from influenza B viruses including both Yamagata and Victoria lineages (Chae, Kim, Kim, et al., 2019). After immunization, positive hybridoma clones (ELISA O.D > 2.0) were selected by ELISA for mRID‐cHA stalk (IAV) and mRID‐cHA (IBV). All 15 of the clones tested were positive for the IAV HA stalk, and 13 out of the 14 clones tested were positive for the IBV HA stalk (100% and 92.9% screening efficiency, respectively). These results demonstrate the Ab repertoire with a clear preponderance toward the target antigen HA stalk over the fusion tag mRID, for both IAV and IBV. We then performed indirect ELISAs using mAbs against the cHA stalk using egg‐derived influenza HA proteins from IAVs (H1N1 and H3N2) and IBVs (Yamagata‐like and Victoria‐like lineages) as coating antigens. The results clearly show that the mAb #5 bound group 1 IAV (H1N1), but not to group 2 IAV(H3N2). Likewise, mAb # 3 specifically bound to two different lineages of IBV with equal efficiency, without any cross‐reactivity to IAV (Figure 6c,d). The availability of group‐specific mAbs will be instrumental in establishing a novel ELISA‐based approach for the quantitation of trivalent seasonal influenza vaccines (Chae, Kim, Kim, et al., 2019; Gravel et al., 2015; Rajendran et al., 2018), pre‐pandemic vaccines, or HA stalk‐based cross‐protective universal vaccine (Erbelding et al., 2018; Jang & Seong, 2014). MAbs that bind to HA‐stalk have been reported to potentiate ADCC (Jegaskanda, Reading, & Kent, 2014). We therefore tested anti‐stalk mAb (IAV and IBV) in the ADCC reporter assay. The results show that mAb # 5 (IAV) potently activated ADCC in IAV infected cells (4.3‐fold) as compared to IBV infected cells (1.3fold) or noninfected control group (1‐fold; Figure 6e). In addition, mAb #3 (IBV) in IBV (B/Yamagata)‐infected cells induced a 4.9‐fold higher ADCC activity compared to cells infected with IAV (H1N1 virus) and non‐mAb treated group (1‐fold; Figure 6f). As a result, the group specific mAbs (IAV and IBV) obtained by immunization with antigens from *E. coli* are expected to recognize the virus infected cells activating the cell‐mediated immune responses.

## 4 DISCUSSION

The quality of research antigens and Abs is central for successful and reproducible immunochemical analyses. Insufficient specificity and high lot‐to‐lot variability of antibodies from commercial sources are not uncommon (Berglund et al., 2008; Slaastad et al., 2011), highlighting the need for improved Abs for research and clinical applications. Although criteria have been suggested as an Ab quality initiative, no obvious areas for improvement have been advanced. The production of high‐quality Abs relies on the availability of pure antigens without immunological complexity or cross‐reactivity. The solubility and conformational status of a given antigen also affects the repertoire of Abs produced, as well as the avidity of the Abs with regard to epitopes (Metzger, 1974, 1978).

**Figure 7:**
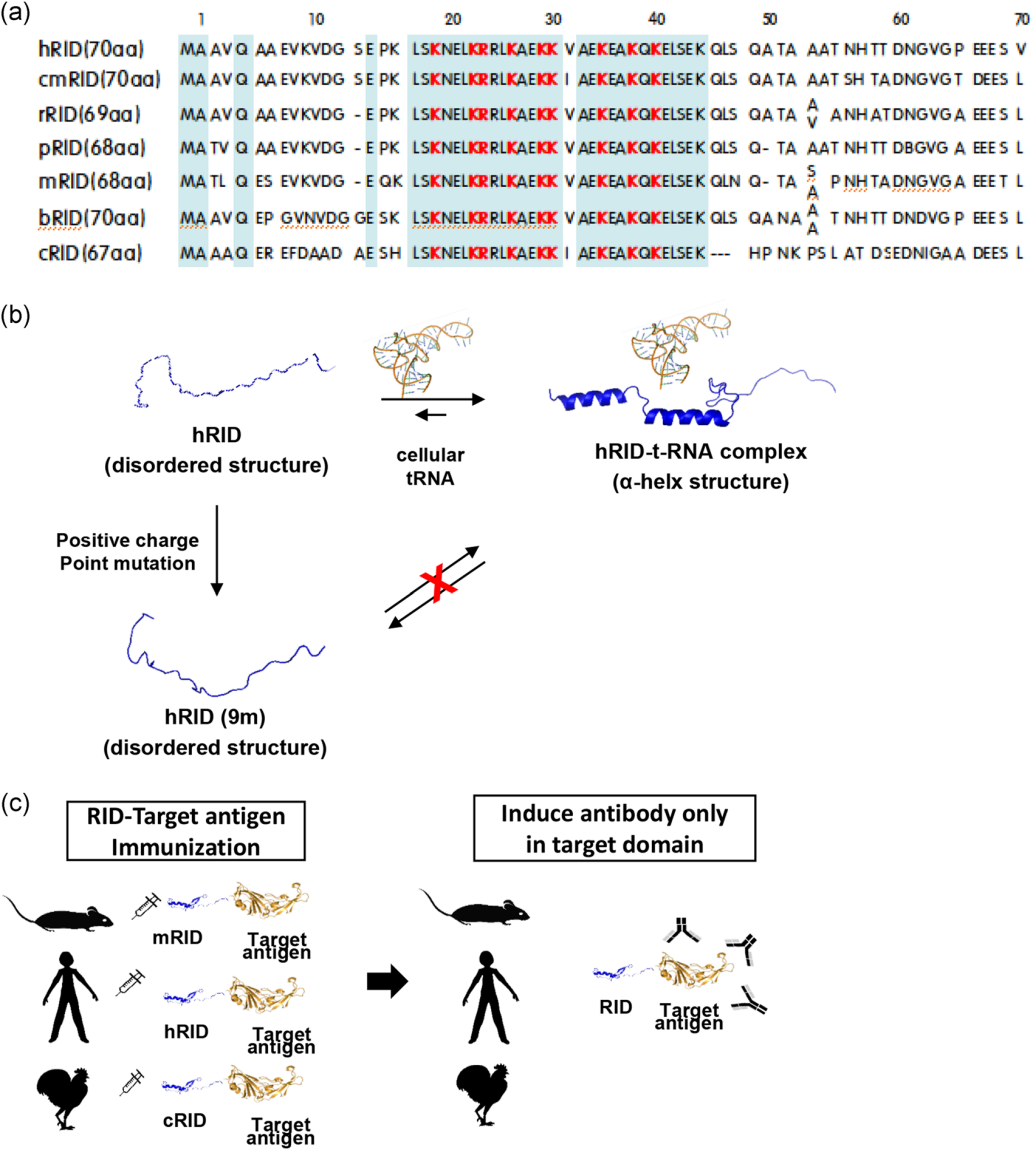
Rationale for the generation of antibodies tailored to human and various animal hosts. (a) Schematic illustration of immune‐tailored design of RID‐fused recombinant antigen. RIDs originating from host animals (mice or chickens) are used as fusion tags for target viral antigens. Immunization with the fusion protein results in an antibody response, predominantly to the target antigen. (b) Structural switching of RID from disorder into alpha‐helix conformation upon tRNA binding. hRID (9 m), which is defective in tRNA binding, is unable to undergo the structural transition and fails to mediate chaperna function. (c) Consensus amino acid sequence of RIDs from various animals. Conserved sequences are represented by shaded blue boxes, and the critical amino acids involved in the alpha‐helical transition resulting from tRNA binding are marked in red. bRID, bat RID; cmRID, camel RID; cRID, chicken RID; hRID, human RID; mRID, mouse RID; pRID, pig RID; RID, RNA‐interacting domain; rRID, rabbit RID; tRNA, transfer RNA [Color figure can be viewed at wileyonlinelibrary.com]

As a step toward improving immunochemicals, we here presented a potentially universal platform for generating soluble antigens from bacterial hosts tailored to a variety of immunized animals for Ab production by harnessing a novel chaperna‐based antigen folding vehicle. Viral antigens are usually produced as insoluble aggregates, and should be solubilized by refolding in vitro before immunization. Solubilization by chemical means using high concentrations of urea or GuCl does not guarantee refolding into the native conformation. Consequently, the loss of conformational epitopes compromises the quality of antibodies directed to refolded antigens (Hagihara, Aimoto, Fink, & Goto, 1993). Using the MERS‐CoV antigen and the influenza HA stalk protein as prototype antigens, we created a potentially universal platform for generating soluble, immunologically relevant recombinant antigens from bacterial hosts. The platform can be tailored to polyclonal (pAb) and mAb production from a variety of animal hosts (Figure 7c). The approach is to harness an RNA‐dependent chaperone; fusion of the target antigen of interest with an RNA‐ docking tag derived from an immunized animal that enables interaction with cellular RNAs, which executes chaperone activity (Figure 1c). The depletion of RNAs by RNase A treatment produced insoluble aggregates of RID fusion proteins (Figure 2a), confirming the crucial role of RNAs in retaining solubility (Choi et al., 2008; J. M. Kim et al., 2017; Son et al., 2015; Yang et al., 2018). The RNA‐binding mutants also enabled the expression of soluble fusion proteins (Figure S2), but predominantly as soluble aggregates that lacked proper conformation, as deduced from various biophysical properties: the binding to NTA column (Figure S3), the elution profile in SEC (Figure S4), and the biological function (binding to the cellular receptor hDPP4; Figure 3c). Harnessing a novel chaperna function, we successfully prepared both the RBD and the HR2 domains from the MERS‐CoV spike protein as soluble and biologically/immunologically relevant conformation by various criteria of biophysical properties (Figures 1b, S3 and S4).

MERS‐CoV is a potentially pandemic virus that has continued to spread among the human population since 2012 with high mortality. No specific and cost‐effective therapies are currently available, but diagnostic kits may help manage the viral infection (Chan et al., 2009; Fukushi et al., 2018). Immunochemical assays using recombinant viral proteins are one of the most effective means of sero‐diagnosis of viral infections. Herein, we successfully produced MERS‐CoV spike protein domains using a chaperna folding vehicle that efficiently generates pAbs and mAbs for the serodiagnosis of MERS‐CoV infections. Previously, only the spike protein or the RBD have been primarily used as diagnostic antigens (Chan et al., 2009; Fukushi et al., 2018; Lee, Ko, Jung, & Nam, 2017). However, the RBD of the virus is frequently mutated, probably as a result of host immune pressure (Kleine‐Weber et al., 2019; Rockx et al., 2010; Tang et al., 2014). Therefore, the inclusion of an additional conserved HR2 antigen (Figure 5a) would establish a highly accurate diagnostic tool for double‐checking for MERS‐CoV infection in humans. Because MERS‐CoV is a potentially zoonotic agent, the serodiagnosis could be extended to animals (Kayali & Peiris, 2015; Milne‐Price, Miazgowicz, & Munster, 2014; Sharif‐Yakan & Kanj, 2014) by utilizing the universal platform tailored to immunized host animals (Figure 7a). The diagnostic platform could be further applied to other emerging viral infections including Corona‐19 (Jin et al., 2020; Singhal, 2020; Zhang et al., 2020).

The circulation of influenza viruses among the human population, and the presence of animal reservoirs of viruses continue to threaten seasonal outbreaks. The HA stalk protein and the specific mAbs generated in the present study are relevant to the current issues regarding influenza control. First, high priority must be given to a “universal” vaccine that could provide cross‐protection against genetic drift or shift strains (Erbelding et al., 2018; Jang & Seong, 2014). The conserved HA stalk domain represents a novel target antigen for cross‐protection. Recently, a correlation between the Ab response to this conserved domain and protection in humans has been identified. Constant fragment (Fc)‐mediated effector function (e.g., ADCC) represents a major mechanism underlying the broad‐spectrum protection offered by candidate universal vaccines (Jegaskanda et al., 2014; Vella, Rocchi, Resta, Marcelli, & Felici, 1980). We did not perform ADCC experiments against other viruses of group 1 (e.g., H2 or H5). However, ELISA assay did confirm broad‐spectrum binding within the same group (including H2 and H5)(Chae, Kim, Hwang, et al., 2019). Our ability to generate HA stalk antigen for all influenza groups, and the ADCC effector function of mAbs (Figure E and F) are relevant to developing recombinant influenza vaccines for cross‐protection.

Second, the licensing of a seasonal influenza vaccine requires a potency assay for the vaccine from the regulatory authorities. The single radial immunodiffusion method is inaccurate, time‐consuming, and requires the supply of strain‐specific reagents (Hardy et al., 2011; Williams, 1993). MAbs against the conserved HA stalk are specific to influenza HAs within the same group (Figure 6c,d). They are used for the quantitation of individual components into trivalent seasonal vaccines that are independent of the annual supply of strain‐specific immunological reagents (Chae, Kim, Kim, et al., 2019; Kuck et al., 2017; Rajendran et al., 2018). The same approach is being extended to the HA of two lineages of IBVs for the quantitation of quadrivalent influenza vaccines. The present results are therefore directly relevant to the development of immunological potency assays for influenza vaccines and for the next generation of influenza vaccine candidates.

Previous attempts to develop influenza recombinant antigens—for example, the cHA stalk or the globular domains of influenza HA—have not been successful in bacterial expression systems without recourse to chemical refolding processes. Thus, serological or potency assays for MERS‐CoV and influenza viruses have relied on recombinant antigens produced from cell‐free translation systems, or recombinant insect or mammalian cells (Reusken et al., 2013; Yamaoka et al., 2016). Although they produce high‐quality antigens with proper folding, these methods produce low yields and require expensive cell culture systems. Assuming proper folding into the native conformation is ensured, bacterial systems have the distinct advantage of low‐cost production over a much shorter time‐scale. Moreover, the advantages of the present chaperna system are manifold.

First, the system is likely to ensure the proper folding of antigens into their native conformations, as proven with influenza hemagglutinin (Yang et al., 2018). The mRID‐HR2 protein is present as a trimer, as the MERS‐CoV spike protein is present on the viral surface in a trimeric conformation. Chapernas have proven instrumental in the macromolecular assembly of antigens into ferritin‐based nanoparticles (*n* = 24) (Kim et al., 2018). Assembly into the native conformation would be conducive to eliciting Ab responses to conformational epitopes, which have the proven ability to neutralize viruses (Kim et al., 2018; Yang et al., 2018).

Second, it is not necessary to remove the mRID before immunization because fusion tag is nonimmunogenic with regard to the host animal. The use of a fusion protein as an immunogen without the necessity of removing the fusion tag simplifies the purification of the antigen, and the Ab production. Previously available fusion tags for promoting solubility have all been bacteria origin (e.g., MBP, GST) (Davis, Elisee, Newham, & Harrison, 1999; Esposito & Chatterjee, 2006; Kapust & Waugh, 1999; Khow & Suntrarachun, 2012), and the immune dominance of the tag has precluded the use of fusion proteins in Ab production.

Third, the lack of immune dominance of the fusion tag reduces the complexity of the Ab repertoires generated by immunization. In the polyclonal sera, the titers for the target antigens were >1,000‐fold higher than the titer for the mRID tag (Figure 5a). The scarcity to hybridoma clones specific to the fusion tag, with a preponderance to target the MERS RBD or the influenza cHA stalk (IAV and IBV) was also manifest in the mAb screening process (Figure 6 and Table 1). It should be noted that the proteins used for immunization were not of high purity (Figure S3). This apparently resulted in cross‐reactive clones owing to bacterial contaminants in the protein preparations (Figure 6a). MAb production is a multi‐step process comprising immunization, the selection of hybridoma cells, and the screening and characterization of positive clones. The screening process—which focuses on identifying and selecting hybridomas that produce antibodies of appropriate specificity—can be time‐consuming, and requires expensive cell culture reagents. This burden is primarily alleviated by using immunogens of high purity, but this often requires multi‐step chromatography. Even without extensive purification, the use of bacterially produced fusion proteins carrying nonimmunogenic tags with built‐in folding capacity is expected to simplify the process of mAb production.

Fourth, and probably with far‐reaching immunochemical implications, is the fact that the fusion tag can be derived from the animal to be immunized. For instance, cRID fusion protein can be used as an immunogen in chickens without disrupting subsequent cleavage and purification. The N‐terminal appendage of LysRS is conserved in the animal kingdom (Figure 7a), and, according to in silico prediction, is able to switch from a disordered state to an alpha‐helical conformation upon tRNA binding (Figure 7b), which operates the chaperna. Importantly, tRNA synthetases are able to interact with non‐cognate tRNAs with low affinity (Francin et al., 2002; Kwon, Yu, et al., 2018). This enables the nonspecific interaction of tRNAs in the *E. coli* cytoplasm with RIDs of any origin, which results in tRNA interaction‐mediated folding of the RID‐fusion antigen (Figure 1c). The RID sequence reveals an RNA‐dependent structural transition motif that is extremely well‐conserved in various vertebrates (Figure 7c). Thus, the same principle of chaperna activity applies to RIDs from any animal, which provides the rationale for the generation of Abs from the animal host of choice (Figure 7c).

In conclusion, we present a potentially universal platform for producing recombinant antigens from a bacterial host enabled by chaperna vehicle. The module is nonimmunogenic but exerts a built‐in chaperone function with regard to the target antigen, and can be tailored to generate antibodies from the animal host of choice. The platform minimizes the time and costs involved in the screening of mAbs, and expedites the development of immunochemical methods for serodiagnostic and therapeutic applications.

## Supporting information

Supplementary information

Supplementary information

Supplementary information
