# Supplementary material for: Built‐in RNA‐mediated chaperone (chaperna) for antigen folding tailored to immunized hosts
Source: Biotechnol Bioeng. 2020 May 2;117(7):1990–2007. doi: 10.1002/bit.27355 (PMC7262357; doi:10.1002/bit.27355)
Supplement: Supplementary file 1 — Supplementary information [file BIT-117-1990-s002.pdf]

Built-in RNA-mediated Chaperone (Chaperna) for Antigen Folding Tailored to Immunized Hosts

Young-Seok Kim[1], Jongkwan Lim[1], Jemin Sung[1], Yucheol Cheong[1], Eun-Young Lee[2], Jihoon Kim[1], Hana Oh[1], Yeon-Sook Kim[3], Nam-Hyuk Cho[4], Seongil Choi[5], Sang-Moo Kang[6], Jae-Hwan Nam[2] , Wonil Chae[1]* and Baik L. Seong[1]*

[1]Department of Biotechnology, College of Life Sciences and Biotechnology, Yonsei University, Seoul, Republic of Korea

[2]Department of Biotechnology, The Catholic University of Korea, Bucheon, Republic of Korea

[3]Division of Infectious Diseases, Department of Internal Medicine, Chungnam National University School of Medicine, Daejeon, Republic of Korea

[4]Department of Microbiology and Immunology, Department of Biomedical Sciences, Seoul National University College of Medicine, Seoul, Republic of Korea

[5]Department of Biochemistry and Biophysics, Stockholm University, SE-106 91, Stockholm, Sweden

[6]Center for Inflammation, Immunity & Infection, Institute for Biomedical Sciences, Georgia State University, Atlanta, GA, USA

*Corresponding author: Baik L. Seong (Phone number : +822-2123-2885, Fax number : +822-362-7265, email : blseong@yonsei.ac.kr,) and Wonil Chae (Phone number : +822-2123-4296, Fax number : +822-362-7265, E.mail : chaewonil@hanmail.net)

Running title: Chaperna-Mediated Antigen Folding

Supplementary Figures and Tables

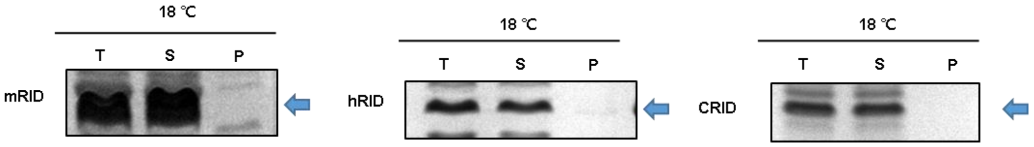

Supplementary Figure 1. Expression levels of chaperna (mRID, hRID, and cRID) produced using an *Escherichia coli* expression system were analyzed by SDS-PAGE.

Supplementary Table 1. Point mutations sites (RNA interaction domain) of mRIDs (wild-type (wt), 2m mutant, and 9m mutant).

**mRID(wt) amino seq**	HMSEQATLQESEVKVDGEQKLSKNELKRRLKAEKKLAEKEAKQKEL<br>SEKQLNQTASAPNHTADNGVGAEEETLDDDDDDSGENLYFQGTGSD<br>IVDKLHHHHHH
**mRID(2m) amino seq**<br>(K23A/K27A)	HMSEQATLQESEVKVDGEQKLSANELARRLKAEKKLAEKEAKQKEL<br>SEKQLNQTASAPNHTADNGVGAEEETLDDDDDDSGENLYFQGTGSD<br>IVDKLHHHHHH
**mRID(9m) amino seq**<br>(K23A/K27A/R28A/K31A/K34A/<br>K35A/K39A/K42A /K44A)	HMSEQATLQESEVKVDGEQKLSANELAARLAAEAALAEAEAAQAEL<br>SEKQLNQTASAPNHTADNGVGAEEETLDDDDDDSGENLYFQGTGSD<br>IVDKLHHHHHH

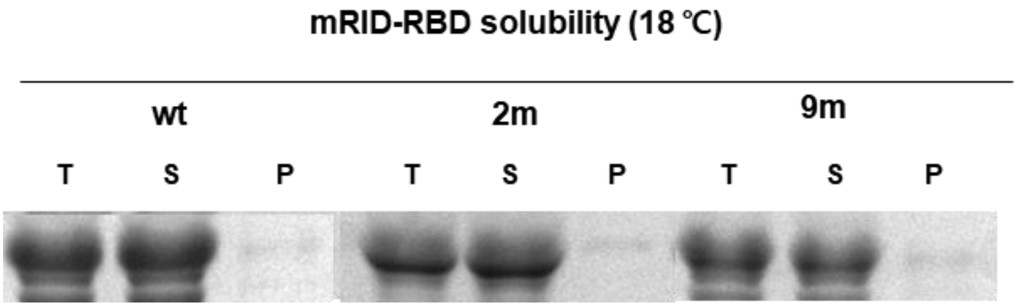

Supplementary Figure 2. Comparison of solubilities of wild-type (wt), 2m mutant, and 9m mutant mRID-RBD molecules. All proteins were expressed at 18°C. The cell lysates were separated into total (T), soluble (S), and pellet (P) fractions by centrifugation. The solubilities of the cell extracts were determined by SDS-PAGE.

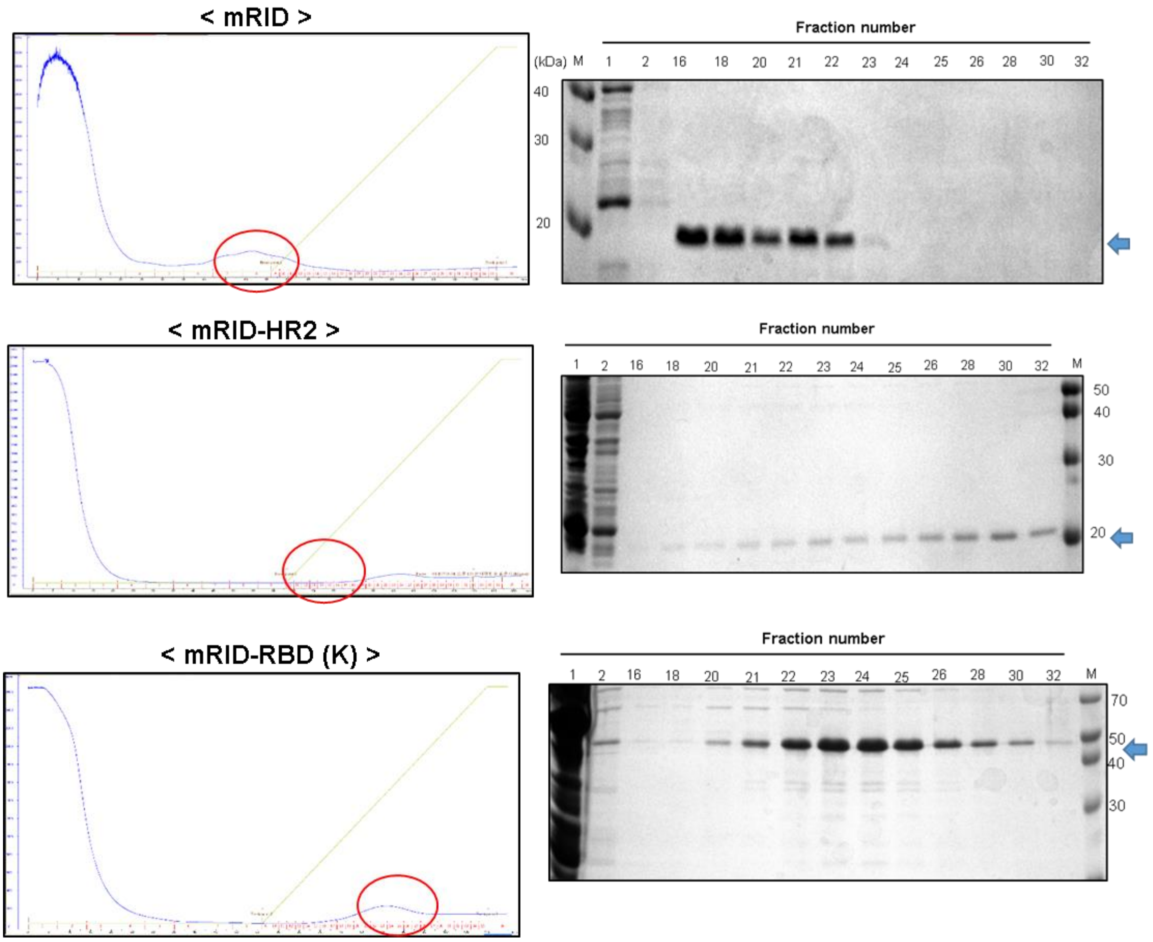

Continued at the next page

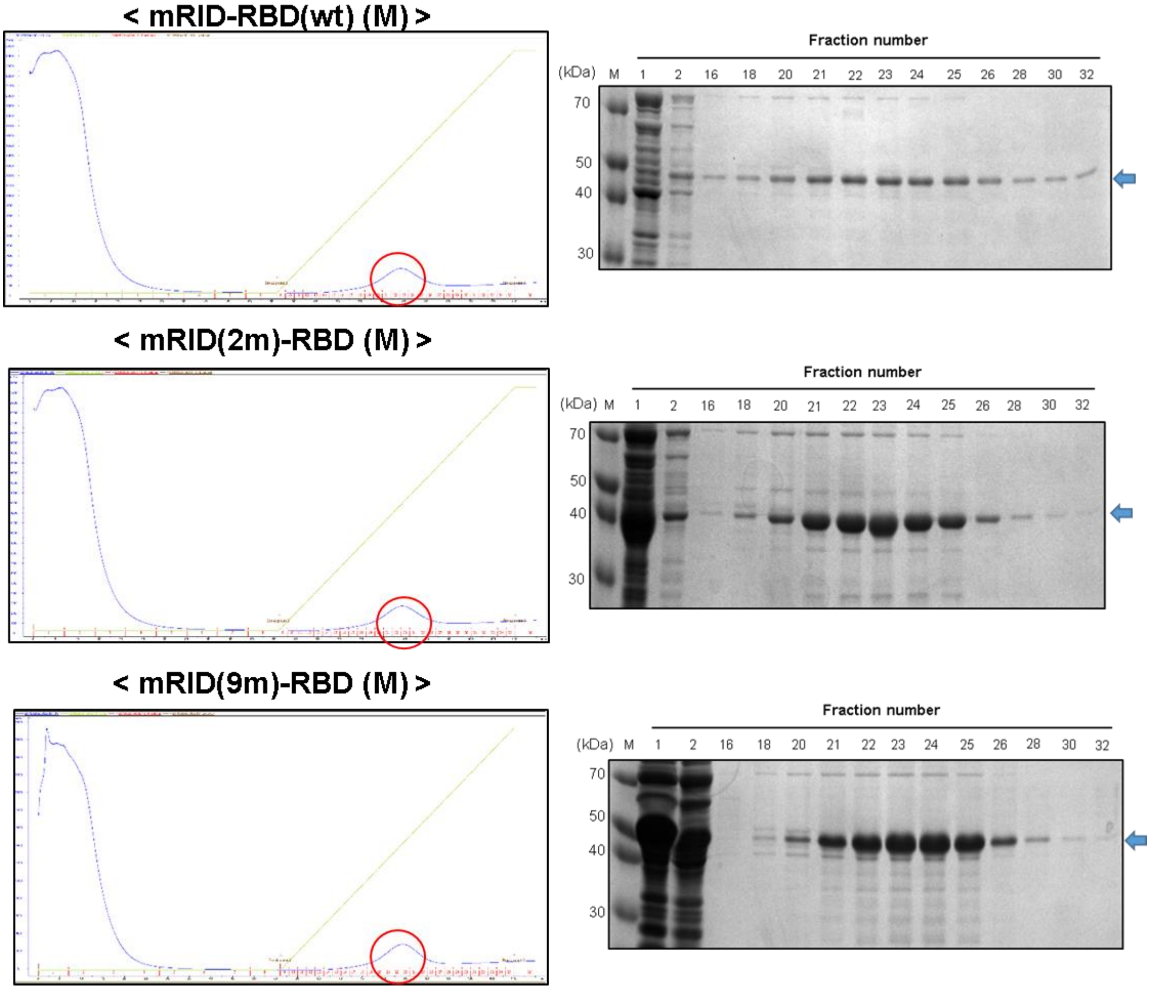

Supplementary Figure 3. Purification profile of proteins obtained by SDS-PAGE. mRID, mRID- RBD (Korea strain), mRID-HR2, mRID(wt)-RBD, mRID(2m)-RBD, and mRID(9m)-RBD were purified using Ni–NTA resin. The blue arrows indicate the purified target proteins.

< mRID(wt)-RBD >

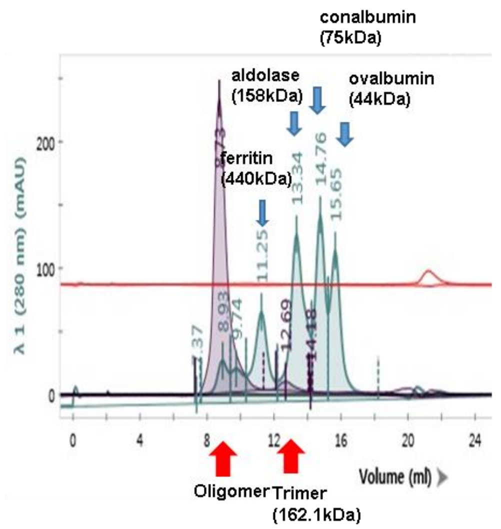

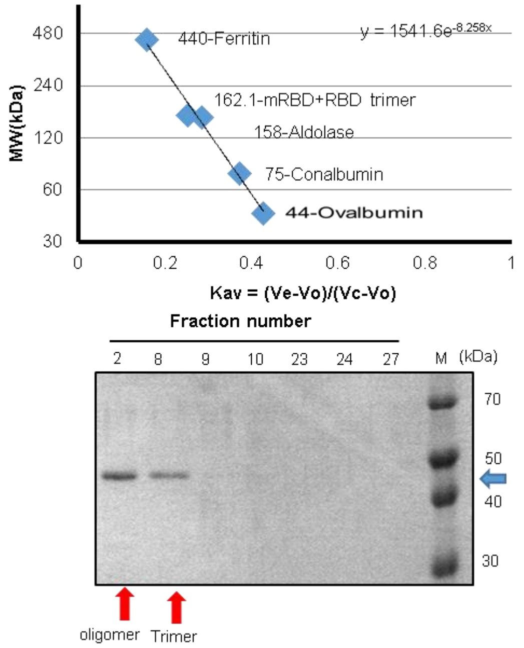

< mRID(9m)-RBD >

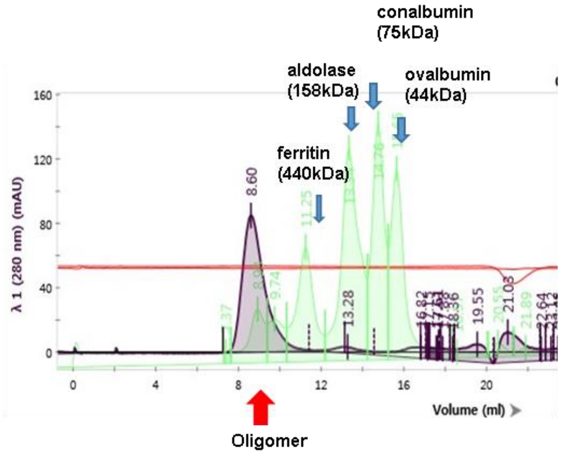

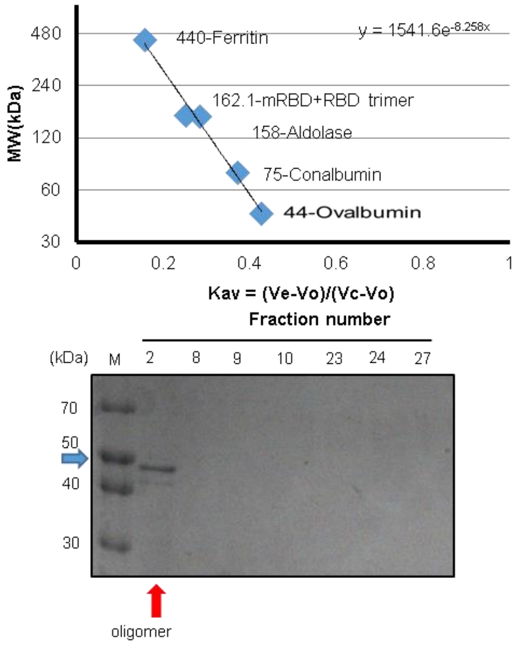

Continued at the next page

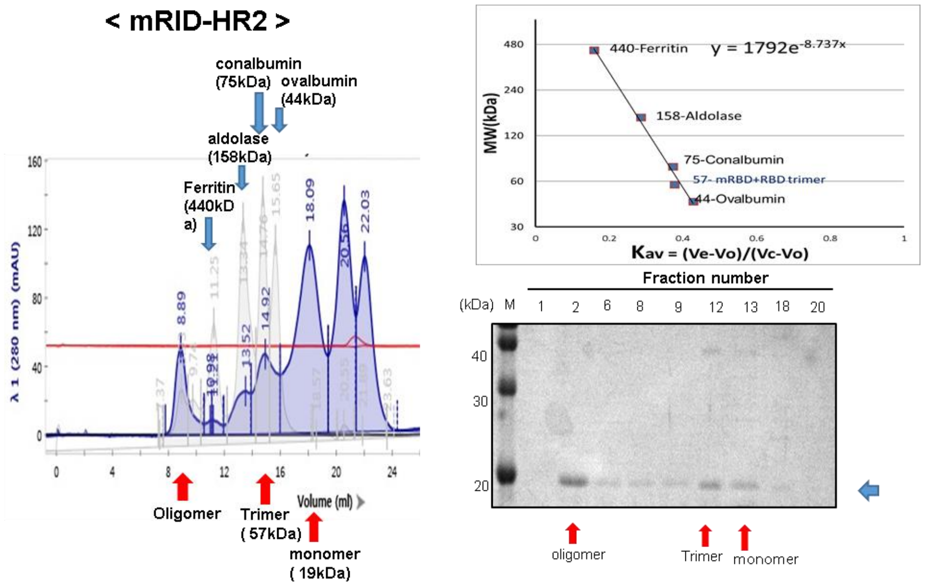

Supplementary Figure 4. Size exclusion chromatography (SEC) profiles of mRID-fused proteins. Separation of purified mRID-RBD (wild-type (wt) and 9m mutant) and mRID-HR2 into oligomers, trimers, or monomers by SEC. The separated proteins were analyzed by SDS-PAGE. The protein sizes were determined using a high molecular weight (HMW) Gel Filtration Calibration Kit and a distribution coefficient (Kav) graph. The blue arrows indicate the purified target proteins.

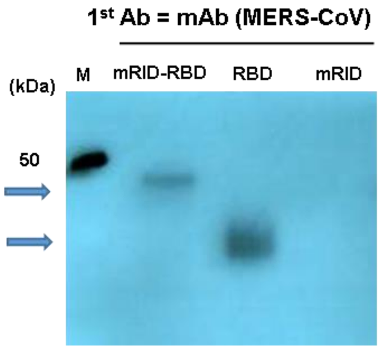

Supplementary Figure 5. The binding of mAb29 to the RBD was assessed by western blot analysis. The blue arrows represent the target proteins. All proteins were purified using a Ni–NTA column.
