# Supplementary material for: Built‐in RNA‐mediated chaperone (chaperna) for antigen folding tailored to immunized hosts
Source: Biotechnol Bioeng. 2020 May 2;117(7):1990–2007. doi: 10.1002/bit.27355 (PMC7262357; doi:10.1002/bit.27355)
Supplement: Supplementary file 2 — Supplementary information [file BIT-117-1990-s001.docx]

**Expression vector construction**

The construction of the pGE-RID(4) vector was based on that of the pGE-LysRS(4) vector (Choi et al., 2008). The mouse-, human-, and chicken-originated RIDs corresponding to amino acids 1 to 69 of the N-terminal of the LysRS were generated by polymerase chain reaction (PCR) and inserted into pGE-LysRS(4) to generate pGE-mRID (the mouse RID), pGE-hRID (the human RID), and pGE-cRID (the chicken RID), respectively. Previous studies have shown that several lysine residues in RID, the N-terminal extension of LysRS, are crucially important for tRNA binding, and the binding-mediated structural transition (Francin et al, 2002; Kwon et al., 2018; Yang et al., 2018). Therefore, Mutant mRIDs were constructed in two forms: mRID(2m) and mRID(9m) by substituting two and nine lysine residues into with alanine, respectively, using site-specific mutagenesis Kit (Elpis-biotech,Daejeon, South Korea; Cat. No. EBT-5001). The amino acid sequences of the mutants are shown in Supplementary Table 1. The genes of the mutant mRIDs were inserted between the *Nde*I and *Kpn*I restriction sites to replace the original mRID in the pGE-mRID(4) vectors. The spike (S) protein of the MERS-CoV sequence (GenBank accession no. AFS88936.1), the RBD of MERS-CoV (residues 367–606 in the S protein), and HR2 (residues 1246–1295) were synthesized by Cosmo Genetech (Seoul, Republic of Korea). We also constructed two types of conserved stalks cHA stalk (IAV) and cHA stalk (IBV), respectively. The cHA stalk (IAV) reflects the consensus sequence from group 1 influenza A viruses (IAVs) by combining high frequency stalk sequences of the H1, H2, H5, and H9 subtypes (Chae et al, 2019). Likewise, the cHA stalk (IBV) sequences reflects the consensus sequences of influenza B viruses including Victoria and Yamagata lineages. The influenza sequence information was analyzed using Vector NTI Advanced® version 11.5 and Seq2logo 2.0, using the sequence library available from the Influenza Virus Resource in the National Center for Biotechnology Information (NCBI- IVR). All the genes were obtained by PCR and inserted between the restriction sites of the pGE-mRID(4) vector.
