# Supplementary figures and images for: Built‐in RNA‐mediated chaperone (chaperna) for antigen folding tailored to immunized hosts
Source: Biotechnol Bioeng. 2020 May 2;117(7):1990–2007. doi: 10.1002/bit.27355 (PMC7262357; doi:10.1002/bit.27355)

**MSERS-CoV S protein sequence**


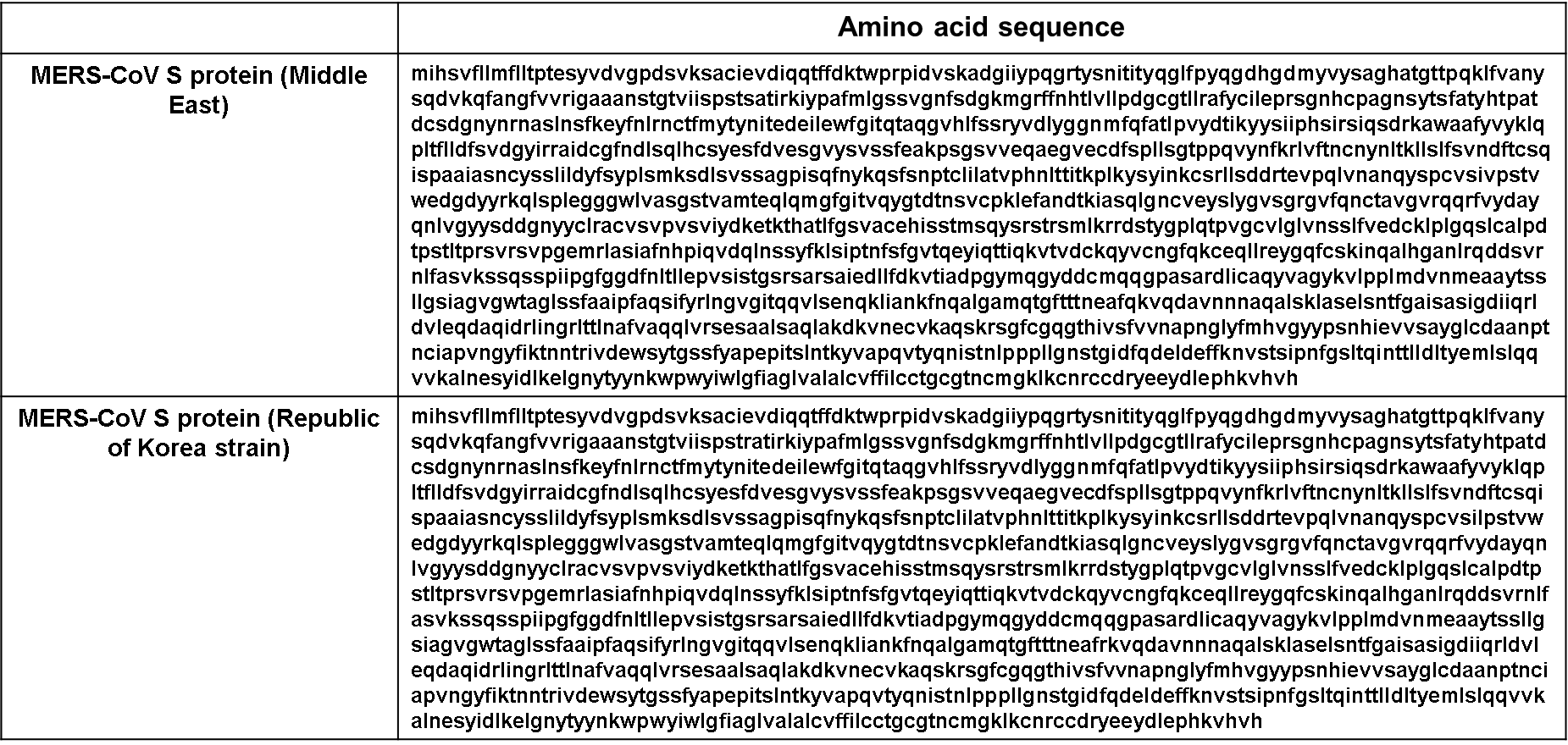

Supplement: Supplementary file 3 — Supplementary information [file BIT-117-1990-s003.docx]
